# Crosstalk Between *Polygonatum kingianum*, the miRNA, and Gut Microbiota in the Regulation of Lipid Metabolism

**DOI:** 10.3389/fphar.2021.740528

**Published:** 2021-10-27

**Authors:** Jincai Dong, Wen Gu, Xingxin Yang, Linxi Zeng, Xi Wang, Jiankang Mu, Yanfang Wang, Fengjiao Li, Min Yang, Jie Yu

**Affiliations:** ^1^ Yunnan Key Laboratory of Southern Medicine Utilization, College of Pharmaceutical Science, Yunnan University of Chinese Medicine, Kunming, China; ^2^ Chenggong Hospital of Kunming Yan’an Hospital, Kunming, China

**Keywords:** microRNA, Polygonatum kingianum Coll. et Hemsl, gut microbiota, lipid metabolism, high-throughput sequencing, correlation analysis

## Abstract

**Objectives:** *Polygonatum kingianum* is a medicinal herb used in various traditional Chinese medicine formulations. The polysaccharide fraction of *P. kingianum* can reduce insulin resistance and restore the gut microbiota in a rat model of aberrant lipid metabolism by down regulating miR-122. The aim of this study was to further elucidate the effect of *P. kingianum* on lipid metabolism, and the roles of specific miRNAs and the gut microbiota.

**Key findings:** *P. kingianum* administration significantly altered the abundance of 29 gut microbes and 27 differentially expressed miRNAs (DEMs). Several aberrantly expressed miRNAs closely related to lipid metabolism were identified, of which some were associated with specific gut microbiota. MiR-484 in particular was identified as the core factor involved in the therapeutic effects of *P. kingianum*. We hypothesize that the miR-484-*Bacteroides/Roseburia* axis acts as an important bridge hub that connects the entire miRNA-gut microbiota network. In addition, we observed that *Parabacteroides* and *Bacillus* correlated significantly with several miRNAs, including miR-484, miR-122-5p, miR-184 and miR-378b.

**Summary:** *P. kingianum* alleviates lipid metabolism disorder by targeting the network of key miRNAs and the gut microbiota.

## Introduction

Lipid metabolism homeostasis requires constant metabolic adjustment, which is partly achieved by regulating the expression of key genes. MicroRNAs (miRNAs) are small non-coding RNAs that regulate gene expression levels by silencing target mRNAs (Hu et al., 2017). and more and more studies suggest that involvement of miRNAs in lipid metabolism and related disorders (Ge et al., 2014; Blasco-Baque et al., 2017; Sliwinska et al., 2017; Sud et al., 2017; Yao et al., 2018).

*Polygonatum kingianum* Coll. et Hemsl (*P. kingianum*) is one of the constituent species in Polygonati Rhizoma, a traditional Chinese medicine formulation. It has various pharmacological activities, such as immuno-stimulatory, anti-aging, blood glucose and lipid regulatory properties (Commission of Chinese Pharmacopoeia, 2020). It has attracted considerable interest in recent years as an effective adjuvant for maintaining the steady state of glucolipid metabolism (Lu et al., 2016; Yan et al., 2017). In a previous study, we found that *P. kingianum* alleviated HFD-fed (HFD)-induced non-alcoholic fatty liver disease (NAFLD) by significantly promoting mitochondrial functions (Yang et al., 2018). It can also alleviate HFD-induced dyslipidemia by regulating many endogenous metabolites in the serum, urine and liver (Yang et al., 2019). There is evidence indicating that fecal miRNAs shape the gut microbiota, and are a potential therapeutic target against metabolic disorders involving dysregulation of the intestinal microbiome (Liu et al., 2016). However, whether how miRNA in liver tissue could affect gut microbiota are still lack of understanding. This reseach focus on imagining whether host miRNAs in liver tissue can also affect gut microbiota. The aim of this study was to explore the miRNAs and the gut microbiota related mechanisms that involved in the ameliorative effect of *P. kingianum* on lipid metabolism disorder.

## Materials and Methods

### Chemicals, Reagents and Materials

Chow diet (62, 26 and 12% calories obtained from carbohydrates, proteins and fats, respectively) was obtained from Suzhou Shuangshi Experimental Animal Feed Technology Co. Ltd. (Suzhou, China). Cholesterol, refined lard and eggs were supplied by Beijing Boao Extension Co. Ltd. (Beijing, China), Sichuan Green Island Co. Ltd. (Chengdu, China) and Wal-Mart Supermarket (Kunming, China) respectively. Simvastatin was purchased from Hangzhou Merck East Pharmaceutical Co. Ltd. (Hangzhou, China), and TRIzol^®^ Reagent from Ambion (Carlsbad, CA, United States). The mircute enhanced miRNA cDNA first strand synthesis kit and the miRNA fluorescence quantitative detection kit were provided by Tiangen Biochemical Technology Co. Ltd. (Beijing, China). D4015 fecal DNA kit was provided by Feiyang Bioengineering Co. Ltd. (Guangzhou, China). Dihydrate oxalic acid was supplied by Sichuan Xiqiao Chemical Co. Ltd. (Sichuan, China) and sodium azide by Amresco (United States). High-purity deionized water was purified using a Milli-Q system (Millipore, Bedford, MA, United States). All other reagents were of analytical grade or higher *P. kingianum* rhizomes were purchased from Wenshan Shengnong Trueborn Medicinal Materials Cultivation Cooperation Society (Wenshan Country, Yunnan Province, China) on April 07, 2016. The samples were authenticated by Professor Jie Yu, and a specimen (No. 8426) was deposited in the Key Laboratory of Preventing Metabolic Diseases of Traditional Chinese Medicine, Yunnan University of Chinese Medicine (Kunming, China).

### Processing of *P. kingianum* Rhizome

The fresh *P. kingianum rhizomes* were processed fellowed our previous studies as well (Gu et al., 2020).

### Preparation of Crude Polysaccharides

The method of preparation of crude polysaccharides fellowed our previous studies as well (Gu et al., 2020).

### Preparation of Total Polysaccharide and High Molecular Weight Polysaccharide Fraction

The method of preparation oftotal polysaccharide (PS) and high molecular weight polysaccharide fraction (PSF) fellowed our previous studies as well (Gu et al., 2020).

### Preparation of Water Extract

The dried *P. kingianum* rhizomes were pulverized, weighed and extracted with 10-, 6-, 4-times volumes of distilled water for 1 h, 40 and 30 min respectively. All water extracts were combined, concentrated under reduced pressure (50 times), frozen to −80°C and then lyophilized (SIM International Group Co. Ltd., Newark, DE, United States). The lyophilized powder was ground, desiccated and stored for later use.

### Quantitative Analysis of PS, PFS, PWE

The method of quantitative analysis of PS, PFS fellowed our previous studies as well (Gu et al., 2020). The tested sample of total saponins in PWE was prepared and tested according the previous method (Lu et al., 2016). PWE lyophilized powder 2.50 g was accurately transferred to 10 ml dissolved, then the tested sample of total polysaccharide in PWE was prepared and tested according the method.

### Induction of Lipid Metabolism Disorder and Treatment Regimen

Feeding and modeling of HFD-rats fellowed our previous studies as well (Gu et al., 2020). In order to study the therapeutic effect of *P. kingianum* on HFD-rats, we refer to the dosage in the literature (Zhao et al., 2015). The middle dose of PS, PSF and PWE materials for rats was set for 240 mg/ kg, the high and low doses are converted correspondingly by 2 times and 0.5 times. Simvastatin (SIM) (1.8 mg/ kg) was administered as a positive control. The rats were randomized into the following groups (*n* = 10 each) and treated with the suitable drug/placebo for 14 weeks (excluding the CON group) *via* the intragastric route (Table 1).

**TABLE 1:** Rats are randomly grouped and dosing status (*n* = 10 each).

No	Drug treatment groups	Dosage (g/kg/d)	Diet
1	Normal Control (NC)	−	Chow Diet
2	Model	−	HFD
3	PSF.L (low-dose PSF)	120 mg/ kg/ d	HFD
4	PSF.M (medium-dose PSF)	240 mg/ kg/ d	HFD
5	PSF.H (high-dose PSF)	480 mg/ kg/ d	HFD
6	PS.L (low-dose PS)	120 mg/ kg/ d	HFD
7	PS.M (midium-dose PS)	240 mg/ kg/ d	HFD
8	PS.H (high-dose PS)	480 mg/ kg/ d	HFD
9	PWE.L (low-dose PWE)	120 mg/ kg/ d	HFD
10	PWE.M (medium-dose PWE)	240 mg/ kg/ d	HFD
11	PWE.H (high-dose PWE)	480 mg/ kg/ d	HFD
12	Positive Control (simvastatin, SIM)	1.8 mg/ kg/ d	HFD

At the end of the 14 weeks, rats were anesthetized intraperitoneally using 1% pentobarbital sodium and euthanized by cervical dislocation. Then, the liver were immediately homogenized in TRIzol (Invitrogen, Carlsbad, CA, United States), snap frozen in liquid nitrogen and stored at−80°C. All reasonable efforts were made to minimize animal suffering.

## MiRNA Sequencing

### Liver RNA Isolation

Three liver tissue samples from each group (upper left anterior lobe of liver) were used for total RNA isolation according to the manufacturer’s protocol. The integrity and purity of the isolated RNA were determined by 1% agarose gel electrophoresis and a NanoPhotometer^®^ (IMPLEN, CA, United States) respectively. RNA concentration was measured using Qubit^®^ RNA Assay Kit in the Qubit^®^ 2.0 Flurometer (Life Technologies, CA, United States). The quality of the samples was further assessed using the RNA Nano 6000 Assay Kit of the Agilent Bioanalyzer 2,100 system (Agilent Technologies, CA, United States) to ensure suitability for high-throughput sequencing.

### Library Preparation

RNA sequencing libraries were generated with 3 μg total RNA per sample using NEBNext ^®^ Multiplex Small RNA Library Prep Set for Illumina^®^ (NEB, USA.) according to the manufacturer’s instructions, and index codes were added to attribute sequences to each sample. The NEB 3′ SR adaptor was directly ligated to the 3′ end of miRNA, siRNA, and piRNA, and the free adapter sequences were hybridized to SR RT primer in order to prevent formation of adaptor-dimers. The 5′ SR adaptor was then ligated to the 5′ends of the transcripts using T4 RNA Ligase 1. First strand cDNA was synthesized using M-MuLV Reverse Transcriptase (RNase H), and amplified using LongAmp Taq 2X Master Mix, SR Primer for Illumina and index (X) primer. The PCR products were purified on a 8% polyacrylamide gel (100 V, 80 min), and the 140–160 bp long fragments (small noncoding RNA plus the 3′ and 5′ adaptors) were recovered in 8 μL elution buffer. Library quality was assessed on the Agilent Bioanalyzer 2,100 system using DNA High Sensitivity Chips.

### Clustering and Sequencing

High-throughput sequencing was performed by Beijing Nuohe Zhiyuan Technology Co. Ltd. The index-coded samples were clustered on a cBot Cluster Generation System using TruSeq SR Cluster Kit v3-cBot-HS (Illumia) according to the manufacturer’s instructions. The library was sequenced on an Illumina Hiseq 2,500 platform and 50bp single-end reads were generated.

## MiRNA Sequencing Data Analysis

### Quality Control

Raw reads in the fastq format were processed through custom perl and python scripts, and the clean reads were obtained by removing low quality reads containing poly-N, 5′ adapter contaminants, lacking 3′adapter or the insert tag and containing poly A/T/G/C. The Q20, Q30, and GC-content of the raw reads were also calculated. Clean reads of a certain range of length were selected for downstream analyses. The small RNA tags were mapped to the reference sequence using Bowtie without mismatch to analyze their expression levels and distribution in the former.

### Known miRNA Alignment

The mapped small RNA tags were used to screen for known miRNAs using mirdeep2, a modified version of miRBase20.0, and srna-tools-cli was used to draw the secondary structures. Custom scripts were used to obtain the miRNA counts and base bias on the first position of identified miRNAs of certain lengths, and on each position of all identified miRNAs. The tags originating from protein-coding genes, repeat sequences, rRNA, tRNA, snRNA, and snoRNA were removed by mapping to RepeatMasker, Rfam database and species specific data.

### Novel miRNA Prediction

Novel miRNAs were predicted using the miREvo and mirdeep2 programs on the basis of secondary structure, Dicer cleavage site and the minimum free energy of the small RNA tags unannotated in the former steps. Custom scripts were used to analyze these identified miRNAs as described in the previous section.

### MiRNA Editing, Family Analysis and Target Gene Prediction

The miRNAs with base edits in the seed region were detected by aligning all the sRNA tags to mature miRNAs with the allowance of one mismatch. The miRNA families were then identified from known miRNAs of other species using miFam.dat (http://www.Mirbase.org/ftp.shtml), and Rfam families from novel miRNA precursors using Rfam (http://rfam.sanger.ac.uk/search/). Target genes of the miRNAs were predicted by psRobot_tar in psRobot using the RNAhybrid, PITA and miRanda algorithms.

### Identification of Differentially Expressed miRNAs

The miRNA expression levels were estimated in terms of TPM (transcript per million) using established criteria, and normalized as mapped read count/total reads*1000000. The significant DEMs between two conditions/groups were screened using the DESeq R package (1.8.3), with corrected (Benjamini and Hochberg method) P-value < 0.05 and fold change (log2, FC) > 1 as the thresholds.

### GO and KEGG Enrichment Analysis

Gene Ontology (GO) enrichment analysis of the target gene candidates of DEMs was performed using GOseq-based Wallenius non-central hyper-geometric distribution, which can adjust for gene length bias. KEGG database was used to screen for the significantly enriched pathways among the target genes (http://www.genome.jp/kegg/), and the statistical significance was tested using KOBAS.

### Quantitative RT-PCR

The DEMs identified above were validated by qRT-PCR. Total RNA was extracted from three randomly selected liver samples per group (36 in total) using Trizol reagent, and reversed transcribed into cDNA using the Tiangen miRcute miRNA cDNA First-Strand Synthesis Kit (Beijing, China). RT-PCR was performed with 2 μL cDNA template and specific primers (Supplementary Table S1) using the miR-cute miRNA Fluorescence Quantification Kit in the ABI PRISM 7300 Real-Time PCR System (Applied Biosystems, Foster City, CA, United States). The reaction parameters were as follows: initial denaturation at 50°C for 2 min, followed by 40 cycles of 95°C for 10 min and one cycle of 95°C–60°C. Two biological replicates were tested per sample, and each reaction was performed in triplicate. U6 was used as the internal standard. Relative expression levels of miRNAs were measured based on threshold cycle values (ct) as 2-ΔΔct.

### Sequencing of Gut Microbiota

The gut microbiota was sequenced as described previously (Gu et al., 2020). Briefly, DNA was extracted from the frozen stool samples using the Fecal DNA Isolation Kit (United States Omega Bio-Tek Co. Ltd), and the 16s rRNA V4 region was amplified using the 515F-806R primer set. Sequencing libraries were generated using TruSeq DNA PCR-Free Sample Preparation Kit (Illumina, San Diego, CA, United States) according to the manufacturer’s instructions and the index codes were added. The library quality was assessed on a Qubit@ 2.0 Fluorimeter (Thermo Scientific Waltham, Massachusetts, United States) and Agilent Bioanalyzer 2,100 system, and sequenced on an IlluminaHiSeq 2,500 platform by Beijing Nuohe Zhiyuan Bioinformatics Co. Ltd. The 250 bp paired-end reads were clustered into OTUs (Operational Taxonomic Units) with 97% consistency by validating each sample. The OTUs abundance was converted to base log 10 to reduce the distance between samples due to the high abundance of OTUs in some.

### Correlation and Network Analysis

To further understand the relationship between miRNA and gut microbiota in rats with lipid metabolism, the Pearson correlation analysis was performed to determine the correlation between DEMs and gut microbiota, and between DEMs and key OTUs, using *p* < 0.05 and R value >0.5 as the thresholds. The Cytoscape software was used to construct and visualize the biological networks between the above pairs.

### Statistical Analysis

The data were analyzed by SPSS 16.0 statistical software (IBM, Armonk, NY, United States), and expressed as mean ± SD. *p* < 0.05 was considered statistically significant. Pearson correlation coefficient was used to determine the strength of correlation between variables as follows: 0.8–1.0–very strong correlation; 0.6–0.8–strong correlation; 0.4–0.6–moderately relevant; 0.2–0.4–weak correlation; 0.0–0.2–very weakly correlated or uncorrelated.

## Results

### Quantitative Analysis Results of PS, PFS, PWE

The specific results of quantitative analysis about PS and PSF as reported in our previous studies as well (Gu et al., 2020). The content of total saponins and polysaccharide in PWE were 2.19 and 1.71% respectively.

### Novel miRNAs Were Identified in the Liver Tissues

The miRNAs dysregulated by HFD feeding were identified in the liver samples using high-throughput sequencing, and as shown in Supplementary Figure S2, the error rate of all but the first five bases was lower than 0.5. In addition, the Q30 score for each sample was not less than 94.02% (Supplementary Table S3), the total number of bases G and C represented the quality of sequencing data. Total G and C percentages were higher than 48.17%, indicated that the sequencing quality was satisfied. After removing low-quality reads, the resulting clean reads accounted for more than 93.58% of all sequences (Supplementary Table S4). The length of the miRNA sequences ranged from 20 to 24 nucleotides, accounting for more than 80% of the pure sequence (Supplementary Figure S3). The 22 nucleotides-long miRNAs were predominant, accounting for about 30% of all sequences, followed by those with 23 and 21 nucleotides. Bowtie analysis indicated that at least 87.07% of the sequences were aligned with the reference genome (Supplementary Table S5). Furthermore, the number of known miRNAs in the sequencing results was relatively high, the proportion of rRNA/tRNA/snRNA/snoRNA was relatively low, as were the number and proportion of positive and negative chains in the exons/introns (Supplementary Table S6). Taken together, the sequences obtained were enriched in miRNAs.

The above-mentioned reads that mapped to the reference sequence were compared with a specified range of sequences in miRBase, and 613 known mature miRNAs and 438 precursors were identified that matched the secondary structure of partially known miRNAs (Supplementary Figure S4). Furthermore, U was the most common first base of miRNAs with lengths of 18–25 nucleotides followed by A, while G and C were less frequent (Supplementary Figure S5). The base distribution of each sample is shown in Supplementary Figure S6; the most and least common first bases were U and G respectively. In addition, 137 mature miRNAs and 142 hairpins matched the secondary structure of partially predicted novel miRNAs (Supplementary Figure S7), which showed similar base preference as the known miRNAs (Supplementary Figures S8, 9).

### Differential miRNA Screening

A total of 135 DEMs–including 70 upregulated (UP) and 65 downregulated (DOWN) miRNAs–were identified in the untreated HFD-fed model versus all other groups (Table 2; Supplementary Figure S10). The expression levels of the significant DEMs are summarized in Supplementary Table S7.

**TABLE 2:** Differentially expressed miRNAs in MOD versus other groups.

No	Groups	Diff	Up	Down
1	MOD *vs.* con	14	10	4
2	MOD *vs.* SIM	9	5	4
3	MOD *vs.* PSF_L	23	9	14
4	MOD *vs.* PSF_M	28	17	11
5	MOD *vs.* PSF_H	10	4	6
6	MOD *vs.* PS_L	5	3	2
7	MOD *vs.* PS_M	7	4	3
8	MOD *vs.* PS_H	7	2	5
9	MOD *vs.* PWE_L	10	3	7
10	MOD *vs.* PWE_M	14	10	4
11	MOD *vs.* PWE_H	8	3	5
12	TOTAL	135	70	65

**FIGURE 1:**
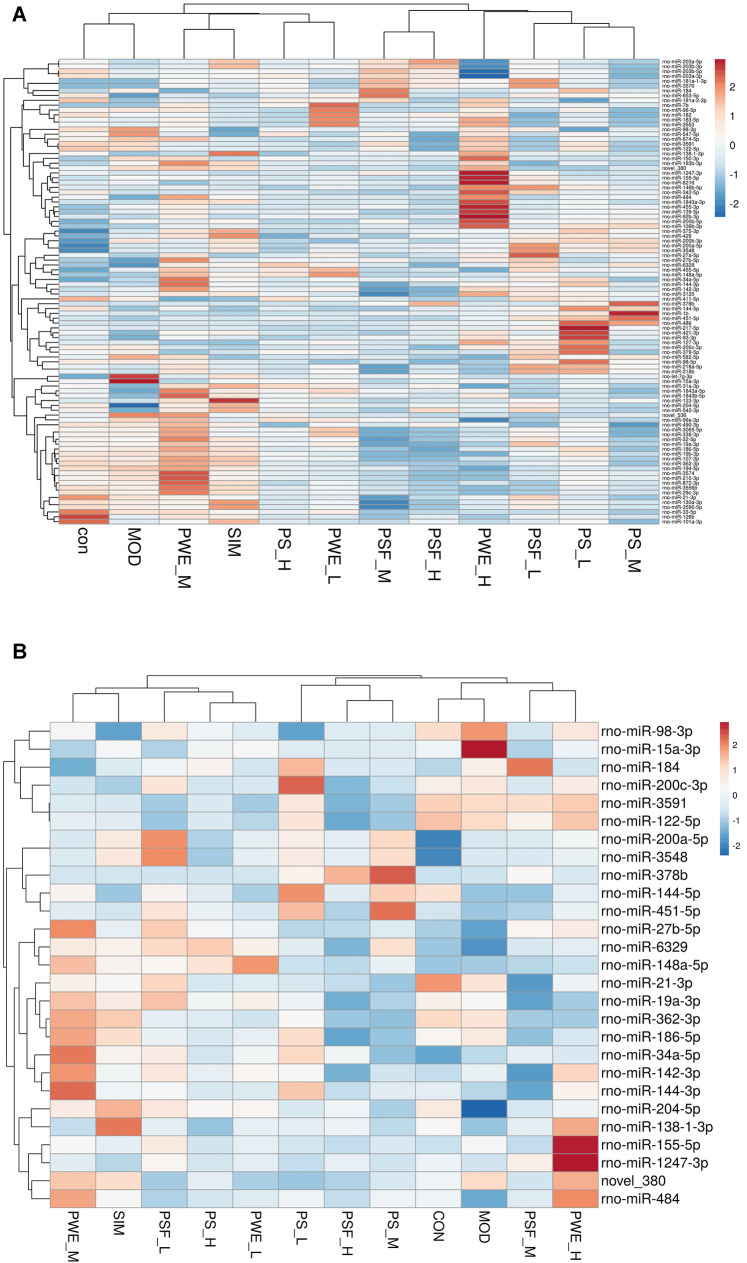
**(A)**. Clustering maps of 135 DEMs **(B)**. Clustering maps of 27 DEMs. The abscissa represents the sample and ordinate DEM. Red and blue blocks respectively indicate the high and low expressing miRNAs.

**FIGURE 2:**
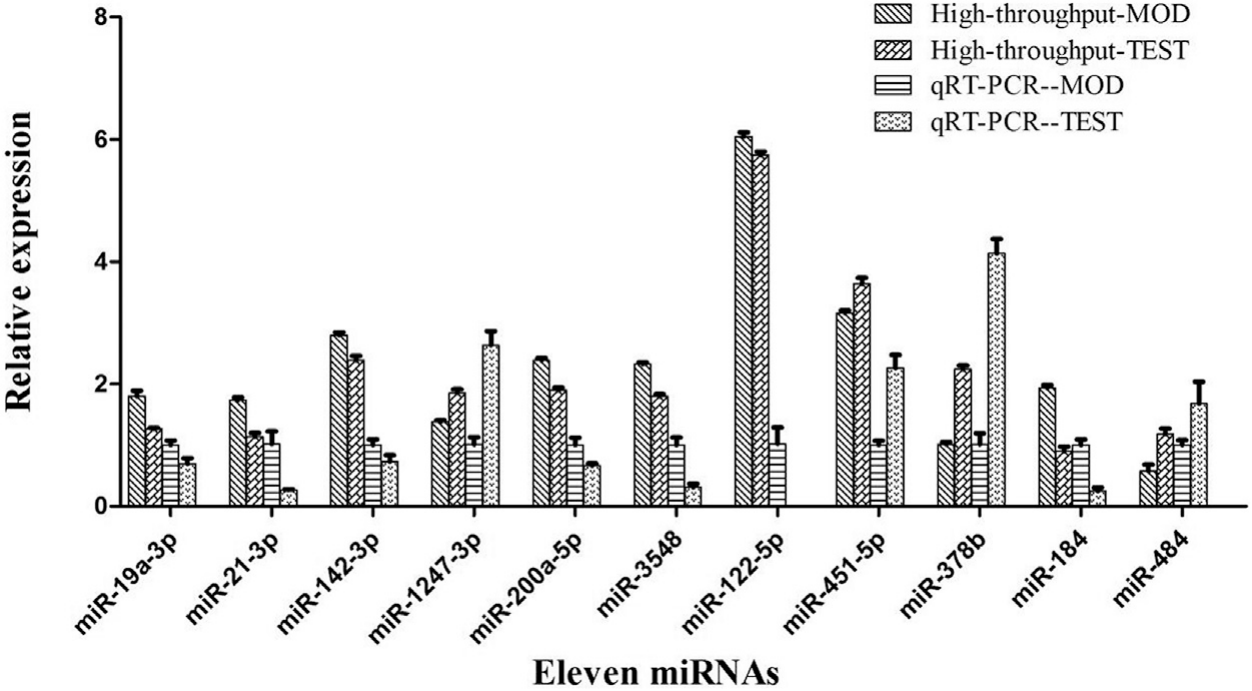
High-throughput sequencing and qRT-PCR of 11 miRNA expression levels.

Hierarchical cluster analysis of the 135 DEMs classified the samples into two categories. The PSF.L, PSF.M, PSF.H, PS.L, PS.M, and PWE.H samples were clustered in one category, indicating the effect of PSF and PS on miRNA levels was largely independent of dosage. Furthermore, the presence of PWE.M and SIM in one cluster indicated that medium-dose PWE had similar effects as simvastatin (Figure 1A). Twenty-seven DEMs with more than two replicates were further screened and subjected to cluster analysis. As shown in Figure 1B, CON, MOD, PSF.M, and PSF.H groups were clustered into one category, and the remaining including PS.H and PWE.L, PWE.M and SIM into the second category, which indicated similar regulatory effects on these DEMs. To verify the accuracy of DEMs identified by high-throughput sequencing, 11 DEMs with two replicates and log2 FC > 1 were further screened by comparing the TEST group (Supplementary Table S8) with the MOD group. As shown in Figure 2, the qRT-PCR results of all DEMs were consistent with that of high-throughput sequencing, indicating reliability of the latter.

### MiRNA Target Gene Prediction

A total of 16,038 targets were predicted for the 135 DEMs using RNAhybrid, PITA, and miRanda (Supplementary Table S9). As shown in Supplementary Table S10, the biological functions of the target genes were diverse, and most of them participate in metabolic pathways of glucose, lipids and nucleotides (*p* < 0.05). KEGG analysis further showed a significant enrichment in type 2 diabetes, insulin secretion and glycerolipid metabolism pathways (Supplementary Figures S11A-C).

### Regulatory Effect of *P. kingianum* on Gut Microbiota in Rats With Lipid Metabolism

*P. kingianum* administration significantly altered the abundance of 29 gut microbes (Supplementary Table S11), which differed in their relative composition across the groups. Similar results were obtained in a previous study as well (Gu et al., 2020). The most significant changes were seen in Roseburia, *Bacteroides* and *Lactobacillus*. *P. kingianum* extracts increased the relative abundance of Roseburia compared to that in the untreated HFD-fed rats. Furthermore, except for PSF.L, the other extracts/dosages increased the abundance of *Bacteroides*, and all extracts significantly reduced the relative abundance of *Lactobacillus*. The three genera are closely related to lipid metabolism (Chen et al., 2015; Dalziel et al., 2017; Huang et al., 2019). The 50 most significantly altered OTUs were screened to further verify the relationship between miRNAs and gut microbiota after *P. kingianum* administration.

### Correlation Between DEMs and Gut Microbiota

**FIGURE 3:**
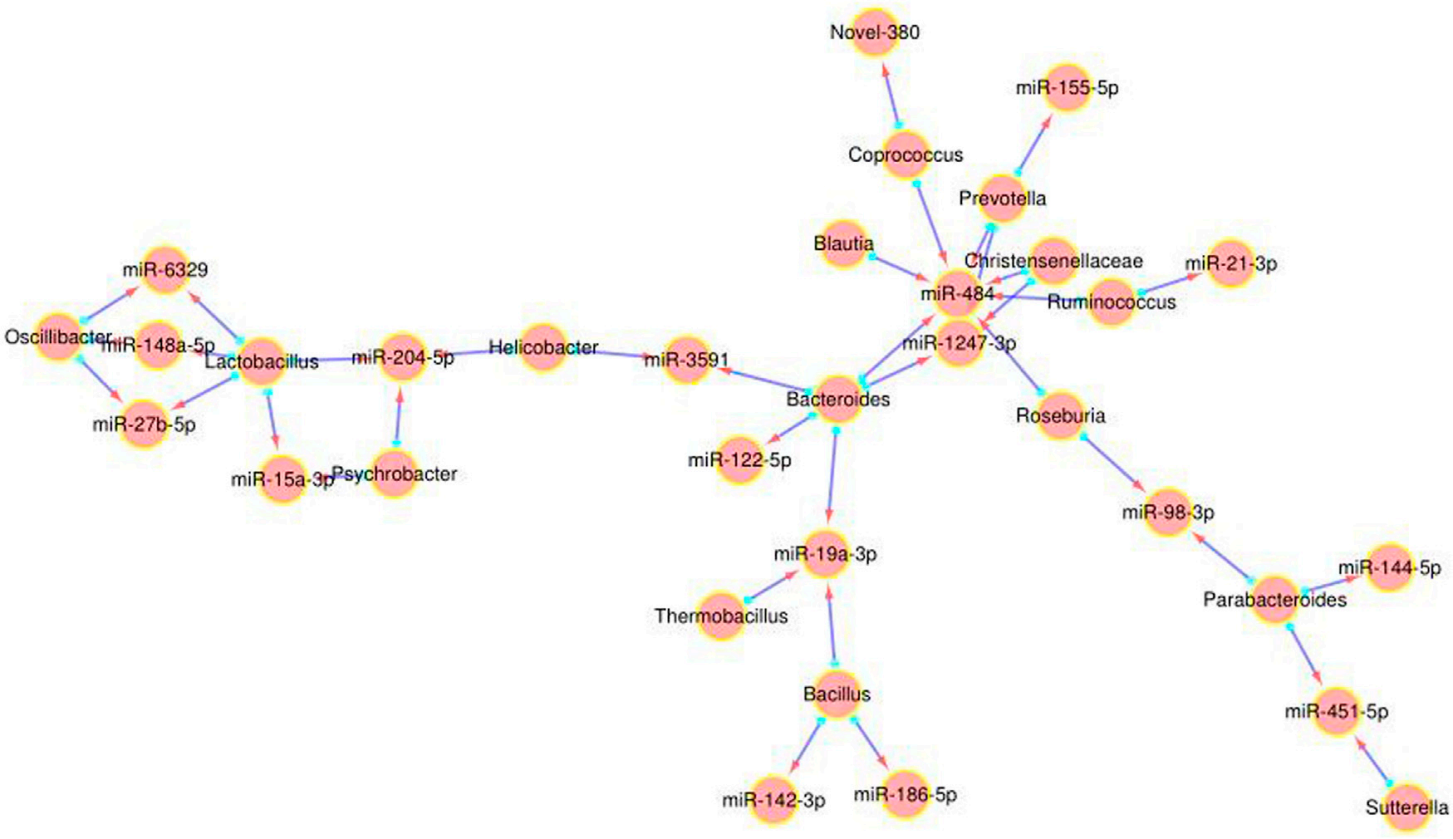
Network analysis of DE miRNA and gut microbiota interactions. Red arrow indicates the miRNA, and the blue origin the intestinal microbes.

**FIGURE 4:**
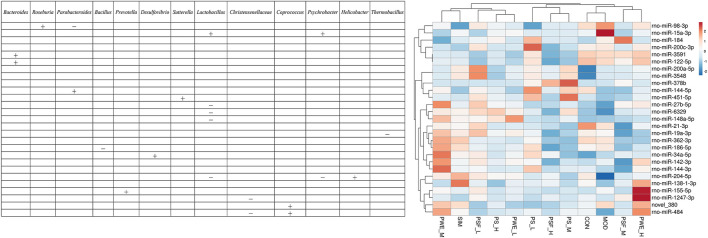
Correlation between significantly altered miRNAs and significantly altered gut microbiota “ + ” Stands for positive correlation, “ − ” stands for negative correlation.

We further analyzed the correlation between the 27 DEMs and the 29 most significantly altered gut microbes, and found that some DEMs were correlated with specific genera in the *P. kingianum*-treated animals (Figure 4). Network analysis further showed that miR-484, miR-204-5p and miR-19a-3p were most significantly correlated with the gut microbiota (Figure 3). As shown in Figure 3; Figure 4 miR-484 was positively correlated with *Bacteroides*, *Roseburia*, *Blautia*, *Prevotella* and *Coprococcus*, and negatively with *Ruminococcus* and *Christensenellaceae*. In fact, miR-484 and *Bacteroides* were the hub that connected the entire network. Furthermore, miR-204-5p showed positive correlation with *Helicobacter*, and negative correlation with *Lactobacillus* and *Psychrobacter*. MiR-19a-3p was negatively correlated with *Bacteroides*, *Bacillus* and *Thermobacillus*. Finally, miR-6329, miR-148a-5p and miR-27b-5p were positively correlated with *Oscillibacter* and negatively correlated with *Lactobacillus*. Taken together, *P. kingianum* affected the interactions between DEMs and the gut microbiota in rats with lipid metabolism disorder.

**FIGURE 5:**
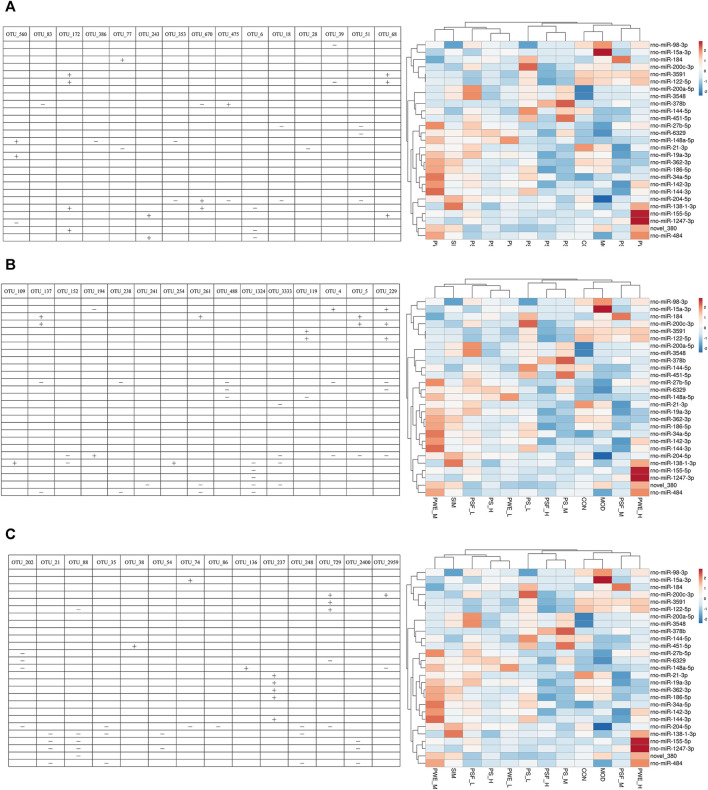
Correlation between significantly altered miRNAs and key OTUs “ + ” Stands for positive correlation, “ − ” stands for negative correlation. **(A)** (OTU_560—OTU_68); **(B)** (OTU_109—OTU_229); **(C)** (OTU_202—OTU_2959).

As shown in Figure 5, 22 DEMs were significantly correlated with 44 key OTUs, of which miR-484, miR-122-5p, miR-184 and miR-378 were closely related to lipid metabolism. MiR-484 was positively correlated with OTU 243 (*Parabacteroides*), and negatively with OTU 6 (*Bacteroidetes*), OTU 137 (*Bacteroidetes*), OTU 238 (*Bacteroidetes*), OTU 261 (*Bacteroidetes*), OTU 1324 (*Bacteroidetes*), OTU 21 (*Firmicutes*), OTU 35 (*Firmicutes*), OTU 248 (*Firmicutes*) and OTU 2400 (*Firmicutes*). MiR-122-5p were negatively correlated with OTU 39 (*Bacteroidetes*) and OTU 88 (*Firmicutes*), and positively with OTU 172 (*Bacteroides*), OTU 68 (*Bacteroidetes*), OTU 119 (*Firmicutes*), OTU 229 (*Firmicutes*) and OTU 729 (*Firmicutes*). In addition, miR-184 were positively correlated with OTU 77 (*Parabacteroides*), OTU 137 (*Bacteroidetes*), OTU 261 (*Bacteroidetes*) and OTU 5 (*Lactobacillus*). Finally, miR-378b were negatively correlated with OTU 83 (*Alloprevotella*) and OTU 670 (*Rikenella*), and positively with OTU 475 (*Parapedobacter*).

## Discussion

### Effect of *P. kingianum* on miRNAs and its Target Genes After HFD-Fed

High-throughput sequencing can identify a large number of genes across the entire genome in a relatively short time, and is therefore an effective tool for discovering and identifying novel miRNAs (Kebschull et al., 2017; Li et al., 2017) It obviates the over-reliance of bioinformatics analysis on species-specific genome-wide information, as well as the interference of the overexpressed cDNA clones in miRNA discovery, and allows identification of novel and differentially expressed miRNAs at different developmental stages and physiological conditions (Wold and Myers, 2008) It is especially suitable for detecting miRNAs alternations following drug administration (Wei et al., 2015; Wang et al., 2019; Zheng et al., 2019). The different extracts of *P. kingianum* significantly altered the liver miRNA profiles of the HFD-fed rats. While the PSF.M significantly increased the relative expression of miR-1247-3p and reduced that of miR-21-3p, PSF.L markedly decreased the expression of miR-122-5p. In addition, PS.M upregulated miR-378b, and PWE.M respectively increased and decreased the levels of miR-484 and miR-184. Some of these miRNAs had been reported to play key regulatory roles in lipid metabolism.

MiR-122 was the first lipid metabolism-related miRNA to be discovered, and is specifically expressed in the liver, accounting for approximately 70% of all liver miRNAs (Filipowicz and Grosshans, 2011). It plays an important role in maintaining normal lipid metabolism (Jopling, 2012), and regulates cholesterol biosynthesis (Krutzfeldt et al., 2005)., fatty acid synthesis and β-oxidation (Elhanati et al., 2016), resulting in lower plasma and liver cholesterol levels and a decrease in fatty acid synthesis (Esau et al., 2006). Consistent with this, PSF significantly downregulated miRNA-122 in the liver, leading to decreased expression of genes involved in downstream lipid synthesis.

MiR-484 is downregulated in pancreatic beta cells in response to hyperglycemic conditions, suggesting that elevated glucose levels in insulin resistance (IR) may affect miR-484 levels in the peripheral blood as well (Tang et al., 2009). Furthermore, miR-484 targets the mitochondrial fission gene Fis1 (Wang et al., 2012), and since mitochondrial fission is increased during diabetes and contributes to circulating insulin levels, downregulation of miR-84 drives the pathogenesis of IR (Wang et al., 2012). PWE.H significantly upregulated miR-484, which is the likely mechanistic basis of its ameliorative effects on lipid metabolism.

### Effect of *P. kingianum* on the miRNAs and Gut Microbiota Network

Studies show the extensive involvement of miRNAs maintaining intestinal homeostasis and the gut microbiota, a complex micro-ecological system that controls host energy metabolism, immune system and inflammatory responses. A previous study identified 16 differentially expressed miRNAs in the cecal tissues of sterile and SPF mice (Singh et al., 2012). Transplanting fecal bacteria from the SPF mice into sterile mice altered the expression levels of 9 miRNAs in the ileum and colon, which in turn affected the expression of at least 700 genes involved in intestinal barrier function and immune regulation (Dalmasso et al., 2011). Consistent with this, the lipid metabolism-related DEMs showed a significant correlation with the altered gut microbiota in the HFD-fed animals, and miR-484, miR-204-5p, and miR-19a-3p were most closely related to changes in the gut microbiota. Correlation analysis of DEMs and key OTUs, along with previous reports on lipid metabolism-related miRNAs, further identified miR-484, miR-122-5p, miR-184, and miR-378b, of which miR-484 was significantly correlated with several gut microbiota such as *Bacteroides*, *Roseburia*, *Ruminococcus*, *Blautia*, *Prevotella*, *Christensenellaceae* and *Coprococcus*. miR-484 was significantly decreased in the HFD-fed rats and restored by *P. kingianum* and simvastatin in this research.

*Bacteroides* is a core intestinal genus (Luis et al., 2018), and shows a lower abundance in the gut of obese individuals (Guo et al., 2008) that is restored following dietary restriction and weight loss. Consistent with this, the abundance of *Bacteroides* was significantly reduced in the HFD-fed animals, and increased after administering different *P. kingianum* extracts. In fact, previous studies have shown a significant regulatory effect of *P. kingianum* polysaccharides on the gut microbiota (Yang et al., 2018; Gu et al., 2020). We found that PS and PSF improved both diabetic symptoms and lipid metabolism. PS and PSF also modulated the gut microbiota composition, abundance and diversity of HFD rats, increased the relative abundance of short chain fatty acid (SCFA) producing bacteria and increased SCFA production, reduced intestinal permeability, relieved gastrointestinal inflammation, and improved lipid metabolism. The abundance of Roseburia was also significantly decreased after HFD feeding, and restored by *P. kingianum* and simvastatin. Likewise, Neyrinck et al. (2011) also detected a lower abundance of *Roseburia* in the gut microbiota of HFD-induced obese mice, which increased significantly after treatment.

Since miR-484 interacted with various gut microbiota, we surmised that it formed the “core” of the regulatory network targeted by *P. kingianum* during lipid metabolism disorder. We hypothesize that the miR-484-*Bacteroides/Roseburia* axis acts an important bridge hub that connects the entire miRNA-gut microbiota network. Further studies should focus on this network, along with miR-148a-5p, miR-27b-5p, miR-6329, miR-204-5p, and miR-19a-3p that interact with multiple intestinal microorganisms. In addition, we observed that *Parabacteroides* and *Bacillus* correlated significantly with several miRNAs, including miR-484, miR-122-5p, miR-184, and miR-378b. The “Parabacteroides-miRNAs” and “Bacillus-miRNAs” networks therefore also warrant further study.

## Summary

In conclusion, *P. kingianum* extracts can alleviate lipid metabolism disorders by targeting the miRNA-gut microbiota network. The causality between miRNAs and gut microbiota, and their interaction play significant role in the preventive and therapeutic effects of *P. kingianum* on lipid metabolism disorders. MiR-484 in particular was identified as the core factor involved in the therapeutic effects of *P. kingianum*. We hypothesize that the miR-484-*Bacteroides*/*Roseburia* axis acts as the core factor involved in the therapeutic effects of *P. kingianum*.

## Data Availability

The data presented in the study are deposited in the National Center for Biotechnology Information repository, accession number PRJNA772872
