# Supplementary material for: Crosstalk Between Polygonatum kingianum, the miRNA, and Gut Microbiota in the Regulation of Lipid Metabolism
Source: Front Pharmacol. 2021 Oct 27;12:740528. doi: 10.3389/fphar.2021.740528 (PMC8578870; doi:10.3389/fphar.2021.740528)
Supplement: Supplementary file 3 [file Presentation1.pdf]

Supplementary Material

1 Supplementary Figures and Tables

1.1 Supplementary Tables

Supplementary Table 1. Differentially expressed miRNA qRT-PCR verified miRNA and primer sequences

Primer Name	Sequence(5'-3')
rno-miR-19a-3p Forward	GCGTGTGCAAATCTATGCAA
rno-miR-19a-3p Reverse	AGTGCAGGGTCCGAGGTATT
rno-miR-21-3p Forward	CGCAACAGCAGTCGATGG
rno-miR-21-3p Reverse	AGTGCAGGGTCCGAGGTATT
rno-miR-122-5p Forward	CGCGTGGAGTGTGACAATGG
rno-miR-122-5p Reverse	AGTGCAGGGTCCGAGGTATT
rno-miR-142-3p Forward	GCGCGTGTAGTGTTTCCTACTT
rno-miR-142-3p Reverse	AGTGCAGGGTCCGAGGTATT
rno-miR-184 Forward	CGCGTGGACGGAGAACTGAT
rno-miR-184 Reverse	AGTGCAGGGTCCGAGGTATT
rno-miR-200a-5p Forward	CGCGCATCTTACCGGACAG
rno-miR-200a-5p Reverse	AGTGCAGGGTCCGAGGTATT
rno-miR-378b Forward	CGCGAGTGGACTTGGAGTC
rno-miR-378b Reverse	AGTGCAGGGTCCGAGGTATT
rno-miR-451-5p Forward	CGCGAAACCGTTACCATTAC
rno-miR-451-5p Reverse	AGTGCAGGGTCCGAGGTATT
rno-miR-484 Forward	GCGTCAGGCTCAGTCCCCT
rno-miR-484 Reverse	AGTGCAGGGTCCGAGGTATT
rno-miR-1247-3p Forward	CGCGGGAACGTCGAGAC
rno-miR-1247-3p Reverse	AGTGCAGGGTCCGAGGTATT
rno-miR-3548 Forward	CGCAGCACTGTCCGGTAA

rno-miR-3548 Reverse	AGTGCAGGGTCCGAGGTATT
U6 Forward	AGAGAAGATTAGCATGGCCCCTG
U6 Reverse	AGTGCAGGGTCCGAGGTATT

Supplementary Table 2. Lipid-related biochemical indicators in rats' blood and liver samples (Mean ± SD, n=7).

Group	Serum TC	Serum TG	Serum HDL-C	Serum LDL-C	Liver TC	Liver TG
CON	1.43±0.12	0.60±0.11***	1.54±0.20***	0.50±0.05	0.77±0.19***	1.46±0.12***
MOD	1.55±0.22	0.89±0.21	1.30±0.08	0.55±0.07	1.17±0.22	2.83±0.71
PSF.L	1.43±0.04	0.96±0.18	1.40±0.06	0.45±0.05*	0.94±0.17*	2.48±0.19
PSF.M	1.53±0.21	1.07±0.21*	1.56±0.17***	0.45±0.13*	0.89±0.14**	2.55±0.21
PSF.H	1.50±0.08	0.84±0.14	1.54±0.19***	0.44±0.05**	1.22±0.20	2.81±0.39
PS.L	1.42±0.18	0.62±0.21**	1.45±0.10*	0.48±0.08	0.94±0.17*	2.45±0.49
PS.M	1.44±0.09	0.67±0.10*	1.45±0.11*	0.49±0.05	0.78±0.15***	1.97±0.08***
PS.H	1.50±0.08	0.69±0.15*	1.47±0.08*	0.51±0.05	0.99±0.07	2.33±0.27*
PWE.L	1.46±0.22	0.87±0.16	1.53±0.21**	0.42±0.09*	1.48±0.30**	2.88±0.47
PWE.M	1.45±0.23	0.78±0.14	1.53±0.22**	0.48±0.08	1.04±0.20	2.28±0.30**
PWE.H	1.51±0.13	0.76±0.16	1.50±0.12*	0.49±0.11	0.95±0.15*	1.20±0.28***
SIM	1.55±0.12	0.76±0.13	1.60±0.12***	0.53±0.05	0.94±0.15*	1.37±0.43***

* indicates a significant difference compared to the model group, $^{*}P < 0.05$, $^{**}P < 0.01$, $^{***}P < 0.001$.

Supplementary Table 3. Data output quality status list

Sample	Reads	Bases	Error rate	Q20	Q30	GC content
A4	12421004	0.621G	0.01%	97.75%	95.24%	49.12%
A5	12747475	0.637G	0.01%	97.95%	95.47%	49.46%
A7	13296982	0.665G	0.01%	97.74%	95.18%	50.27%
B2	12360128	0.618G	0.01%	98.01%	95.64%	49.51%
B3	13058584	0.653G	0.01%	97.66%	95.03%	50.42%
B8	15247335	0.762G	0.01%	97.52%	94.47%	48.75%
C3	14352960	0.718G	0.01%	98.08%	95.81%	48.70%
C7	12720262	0.636G	0.01%	98.13%	95.84%	49.31%
C10	13779509	0.689G	0.01%	98.08%	95.85%	48.54%
D1	12027228	0.601G	0.01%	97.31%	94.17%	48.66%
D2	13012192	0.651G	0.01%	97.70%	95.14%	48.90%
D3	16546825	0.827G	0.01%	97.76%	94.93%	48.72%
E1	11086312	0.554G	0.01%	97.64%	94.93%	48.17%
E6	11829462	0.591G	0.01%	97.31%	94.19%	48.70%
E9	12183363	0.609G	0.01%	97.41%	94.36%	48.77%
F5	11739384	0.587G	0.01%	97.24%	94.04%	48.93%
F7	14832062	0.742G	0.01%	97.62%	94.94%	49.46%
F10	12638910	0.632G	0.01%	97.72%	95.18%	48.79%
G1	12676623	0.634G	0.01%	97.23%	94.02%	49.33%
G2	12916298	0.646G	0.01%	97.26%	94.10%	49.16%
G8	11043558	0.552G	0.01%	97.49%	94.60%	49.45%
H1	13622367	0.681G	0.01%	97.75%	95.23%	48.80%
H4	14490965	0.725G	0.01%	97.85%	95.28%	49.47%
H9	13594326	0.680G	0.01%	97.61%	94.66%	48.71%
I2	13228996	0.661G	0.01%	97.69%	95.12%	49.04%
I4	15247587	0.762G	0.01%	98.41%	96.55%	48.71%
I7	14030572	0.702G	0.01%	98.46%	96.65%	48.88%

J2	13766445	0.688G	0.01%	98.04%	95.66%	49.77%
J6	13506671	0.675G	0.01%	97.71%	95.16%	49.78%
J7	15373809	0.769G	0.01%	98.31%	96.34%	49.22%
K1	14135749	0.707G	0.01%	98.13%	95.91%	48.91%
K5	11535959	0.577G	0.01%	98.33%	96.40%	49.01%
K6	13257891	0.663G	0.01%	98.08%	95.69%	51.80%
L1	15645729	0.782G	0.01%	97.68%	95.09%	49.21%
L9	12913291	0.646G	0.01%	98.06%	95.74%	49.24%
L10	13345779	0.667G	0.01%	98.08%	95.80%	49.77%

Note：
(1) Sample： samples id.
(2) Reads： The original sequence data is counted, and the number of sequencing sequences of each sequencing file is counted in one unit of four behaviors.
(3) Bases： The number of sequencing sequences is multiplied by the length of the sequencing sequence and converted to G.
(4) Error rate： Refers to the sequencing error rate, calculated by the formula 1.
(5) Q20： The base of the Phred value greater than 20 is a percentage of the total base.
(6) Q30： Bases with a Phred value greater than 30 as a percentage of the total base.
(7) GC content： Calculate the sum of the number of bases G and C as a percentage of the total number of bases

Supplementary Table 4. Data filtering situation list.

Sample	Total reads	N%>10%	Low quality	5 adapter contamine	3 adapter null or insert null	With ployA/T/G/C	Clean reads
A4	12421004 (100.00%)	6 (0.00%)	47077 (0.38%)	1724 (0.01%)	582532 (4.69%)	15281 (0.12%)	11774384 (94.79%)
A5	12747475 (100.00%)	1575 (0.01%)	25834 (0.20%)	1065(0.01%)	530824 (4.16%)	39828 (0.31%)	12148349 (95.30%)
A7	13296982 (100.00%)	7(0.00%)	48455 (0.36%)	1441 (0.01%)	785760 (5.91%)	17485 (0.13%)	12443834 (93.58%)
B2	12360128 (100.00%)	1494 (0.01%)	23924 (0.19%)	1003(0.01%)	458162 (3.71%)	24201 (0.20%)	11851344 (95.88%)
B3	13058584 (100.00%)	4 (0.00%)	57440 (0.44%)	2761(0.02%)	464905 (3.65%)	11015 (0.08%)	12522459 (95.89%)
B8	15247335 (100.00%)	4691 (0.03%)	33136 (0.22%)	423(0.00%)	453725 (2.98%)	11945 (0.08%)	14743415 (96.70%)
C3	14353960 (100.00%)	1773 (0.01%)	30813 (0.21%)	531 (0.00%)	457958 (3.19%)	13483 (0.09%)	13848402 (96.48%)
C7	12720262 (100.00%)	1600 (0.01%)	23718 (0.19%)	614 (0.00%)	409989 (3.22%)	19290 (0.15%)	12265051 (96.42%)
C10	13779509 (100.00%)	1703 (0.01%)	26979 (0.20%)	343 (0.00%)	569349 (4.13%)	7775 (0.06%)	13173360 (95.60%)
D1	12027228 (100.00%)	416 (0.00%)	104372 (0.87%)	282 (0.00%)	297642 (2.47%)	6408 (0.05%)	11618108 (96.60%)
D2	13012192 (100.00%)	6 (0.00%)	56766 (0.44%)	534 (0.00%)	358024 (2.75%)	8400 (0.06%)	12588462(96.74%)

D3	16546825 (100.00%)	3097 (0.02%)	15042 (0.09%)	361 (0.00%)	560212 (3.39%)	10505 (0.06%)	15957608 (96.44%)
E1	11086312 (100.00%)	405 (0.00%)	66357 (0.60%)	111 (0.00%)	271624 (2.45%)	2624 (0.02%)	10745191 (96.44%)
E6	11829462 (100.00%)	442 (0.00%)	110822 (0.94%)	303 (0.00%)	378150 (3.20%)	9273(0.08%)	11330472 (95.78%)
E9	12183363 (100.00%)	445 (0.00%)	110251 (0.90%)	412 (0.00%)	387636 (3.18%)	6468 (0.05%)	11678151 (95.85%)
F5	11739384 (100.00%)	442 (0.00%)	97003 (0.83%)	408 (0.00%)	404921 (3.45%)	11861 (0.10%)	11224749 (95.62%)
F7	14832062 (100.00%)	357 (0.00%)	45345 (0.31%)	2872(0.02%)	581680 (3.92%)	12418 (0.08%)	14189390 (95.67%)
F10	12638910 (100.00%)	320 (0.00%)	37795 (0.30%)	603 (0.00%)	392886 (3.11%)	20671 (0.16%)	12186635 (96.42%)
G1	12676623 (100.00%)	479 (0.00%)	108109 (0.85%)	678 (0.01%)	602912 (4.76%)	16634 (0.13%)	11947811 (94.25%)
G2	12916298 (100.00%)	485 (0.00%)	111623 (0.86%)	749 (0.01%)	467450 (3.62%)	18579 (0.14%)	12317412 (95.36%)
G8	11043558 (100.00%)	411 (0.00%)	65508 (0.59%)	370 (0.00%)	394403 (3.57%)	7898(0.07%)	10574968 (95.76%)
H1	13622367 (100.00%)	10 (0.00%)	57329 (0.42%)	630 (0.00%)	478369 (3.51%)	10069 (0.07%)	13075960 (95.99%)
H4	14490965 (100.00%)	1787 (0.01%)	30435 (0.21%)	1135 (0.01%)	496149 (3.42%)	19645(0.14%)	13941814 (96.21%)
H9	13594326 (100.00%)	1697 (0.01%)	28910 (0.21%)	734 (0.01%)	427041 (3.73%)	10452(0.08%)	13125492 (96.55%)
I2	13228996 (100.00%)	14 (0.00%)	53939 (0.41%)	719 (0.01%)	493499 (3.73%)	17861 (0.14%)	12662964 (95.72%)

I4	15247587 (100.00%)	2361 (0.02%)	44480 (0.29%)	698 (0.00%)	385331 (2.53%)	12993 (0.09%)	14801724 (97.08%)
I7	14030572 (100.00%)	2109 (0.02%)	33465 (0.24%)	783 (0.01%)	436204 (3.11%)	10082 (0.07%)	13547929 (96.56%)
J2	13766445 (100.00%)	1667 (0.01%)	26873 (0.20%)	1101 (0.01%)	493111 (3.58%)	12036 (0.09%)	13231657(96.12%)
J6	13506671 (100.00%)	8(0.00%)	64474 (0.48%)	2141(0.02%)	513341 (3.80%)	13724 (0.10%)	12912983 (95.60%)
J7	15373809 (100.00%)	2334 (0.02%)	40429 (0.26%)	962 (0.01%)	576533 (3.75%)	10229 (0.07%)	14743322 (95.90%)
K1	14135749 (100.00%)	1704 (0.01%)	26427 (0.19%)	634 (0.00%)	493985 (3,49%)	11003 (0.08%)	13601996 (96.22%)
K5	11535959 (100.00%)	1701 (0.01%)	31139 (0.27%)	680 (0.01%)	407006 (3.53%)	8764 (0.08%)	11086669 (96.11%)
K6	13257891 (100.00%)	1621 (0.01%)	21821 (0.16%)	1167 (0.01%)	412921 (3.11%)	46797 (0.35%)	12773564 (96.35%)
L1	15645729 (100.00%)	10(0.00%)	51957 (0.33%)	2529(0.02%)	664894 (4.25%)	27934 (0.18%)	14898405 (95.22%)
L9	12913291 (100.00%)	1601 (0.01%)	24133 (0.19%)	905(0.01%)	42661 (3.30%)	17996 (0.14%)	12442040 (96.35%)
L10	13345779 (100.00%)	1602 (0.01%)	26494 (0.20%)	1158(0.01%)	489044 (3,66%)	21224 (0.16%)	12806257 (95.96%)

Note：

(1) Sample：sample id。
(2) total_reads：Count the number of original sequence data.
(3) N% > 10%：The ratio of the number of reads filtered out and the total number of raw reads due to the N content exceeding 10%.
(4) low quality：Due to low quality, the number of copies that are filtered out and their proportion to the total number of raw reads.
(5) 5_adapter_contamine：The number of reads that are filtered out and their proportion to the total number of raw reads due to the 5' joint.

(6) 3_adapter_null or insert_null：The number of reads that are filtered out and their proportion to the total number of raw reads, since there are no 3' connectors or no inserts.
(7) with ployA/T/G/C：The number of reads filtered and their proportion to the total number of raw reads due to the inclusion of plotA/T/G/C.
(8) clean reads：The resulting number of clean reads and their proportion to the total number of raw read

Supplementary Table 5. Alignment with reference genome.

Sample	Total sRNA	Mapped sRNA	"+" Mapped sRNA	" - " Mapped sRNA
A4	9957773 (100.00%)	9171075 (92.10%)	5827771 (58.52%)	3343304 (33.57%)
A5	9929824 (100.00%)	8989999 (90.54%)	5093774 (51.30%)	3896225 (39.24%)
A7	9062665 (100.00%)	8386298 (92.54%)	5508280 (60.28%)	2878018 (31.76%)
B2	9877613 (100.00%)	9058975 (91.71%)	4969564 (50.31%)	4098411 (41.40%)
B3	10903089 (100.00%)	10199924 (93.55%)	7411371 (67.97%)	2788553 (25.58%)
B8	14215131 (100.00%)	13073698 (91.97%)	7264916 (51.11%)	5808782 (40.86%)
C7	11789813 (100.00%)	10265833 (87.07%)	5450452 (46.23%)	4815381 (40.84%)
C10	12544891 (100.00%)	11648895 (92.86%)	6067417 (48.37%)	5581478 (44.49%)
D1	10995598 (100.00%)	10092981 (91.79%)	5821993 (52.95%)	4270988 (38.84%)
D2	11785873 (100.00%)	10980277 (93.16%)	6115128 (51.89%)	4865149 (41.28%)
D3	15162458(100.00%)	13899690 (91.67%)	8161808 (53.83%)	5737882 (37.84%)
E1	10531734(100.00%)	9807608 (93.12%)	3263862 (30.99%)	6543746 (62.13%)
E6	10656185 (100.00%)	9661029 (90.66%)	5337571 (50.09%)	4323458 (40.57%)
E9	10999441 (100.00%)	10171968 (92.48%)	5244069 (47.68%)	4927899 (44.80%)
F5	10285683 (100.00%)	9236454 (90.06%)	5058089 (49.18%)	4205405 (40.89%)
F7	12806146 (100.00%)	11498360 (89.79%)	7307487 (57.06%)	4190873 (32.73%)
F10	11705711 (100.00%)	10878172 (92.93%)	6097979 (52.09%)	4780193 (40.84%)

G1	10488619 (100.00%)	9543915 (90.99%)	5345553 (50.97%)	4632411 (40.88%)
G2	1133087 (100.00%)	10179499 (89.84%)	5547088 (48.96%)	4198362 (40.03%)
G8	10193191 (100.00%)	9000777 (88.30%)	4356897 (42.74%)	4643880 (45.56%)
H1	12344375 (100.00%)	11407697 (92.41%)	5830137 (47.23%)	5577560 (45.18%)
H4	12887729 (100.00%)	11935831 (92.61%)	7129792 (55.32%)	4806039 (37.29%)
H9	12466355 (100.00%)	11588383 (92.96%)	6222783 (49.92%)	5365600 (43.04%)
I2	11859522 (100.00%)	10609054 (89.46%)	5279823 (44.52%)	5329231 (44.94%)
I4	13863420 (100.00%)	12804597 (92.36%)	7061094 (50.93%)	5743503 (41.43%)
I7	12803089 (100.00%)	11548265 (90.20%)	5818854 (45.45%)	5729411 (44.75%)
J2	11703399 (100.00%)	10803163 (92.31%)	6412740 (54.79%)	4390423 (37.51%)
J6	11334709(100.00%)	10483748 (92.49%)	6734790 (59.42%)	3748959 (33.08%)
J7	13656246 (100.00%)	12661944 (92.72%)	6656399 (48.74%)	6005545 (43.98%)
K1	12633606 (100.00%)	11742813 (92.95%)	5912644 (46.80%)	5830169 (46.15%)
K5	10496375(100.00%)	9610236 (91.56%)	5089336 (48.49%)	4520900 (43.07%)
K6	12052339 (100.00%)	10509833 (87.20%)	7829142 (64.96%)	2680691 (22.24%)
L1	12682274 (100.00%)	11549158 (91.07%)	7145029 (56.34%)	4041129 (34.73%)
L9	11170677 (100.00%)	10360595(92.75%)	5237357 (46.88%)	5123238 (45.86%)
L10	11873978 (100.00%)	10925397 (92.01%)	6402049 (53.92%)	4523348 (38.09%)

Note：(1) Sample：sample id。

(2) Total sRNA：The total number of reads obtained for each sample after analysis of "sRNA length screening".

(3) Mapped sRNA： The number of reads and percentages that can be mapped to the reference sequence in the sample reads.
(4) “+” Mapped sRNA： The number of reads and the percentage of the same chain that can be mapped to the reference sequence in the sample reads.

(5) “-” Mapped sRNA： The sample reads mapped to the number of reads in the opposite direction of the reference sequence and the percentage

Supplementary Table 6. sRNA classification statistics table

Types	total	Known-miRNA	rRNA	tRNA	snRNA	snoRNA	repeat	Novel-miRNA	Exon:+	Exon:-	Intron:+	Intron:-	other
A4	9171075	4204528	34267	203919	10172	61383	201227	656	249226	31834	182887	68993	3921983
A4	100.00%	45.85%	0.37%	2.22%	0.11%	0.67%	2.19%	0.01%	2.72%	0.35%	1.99%	0.75%	42.76%
A5	8989999	2778375	44548	298344	24059	79704	436643	407	1092458	51478	361607	61667	3760709
A5	100.00%	30.91%	0.50%	3.32%	0.27%	0.89%	4.86%	0.00%	12.15%	0.57%	4.02%	0.69%	41.83%
A7	8386298	3903214	38102	158595	8552	57769	258959	767	320828	36934	199888	71437	3331253
A7 (percent)	100.00%	46.54%	0.45%	1.89%	0.10%	0.69%	3.09%	0.01%	3.83%	0.44%	2.38%	0.85%	39.72%
B2	9058975	3546089	50416	128554	14240	72761	417005	653	826165	65150	296003	99042	3542897
B2	100.00%	39.14%	0.56%	1.42%	0.16%	0.80%	4.60%	0.01%	9.12%	0.72%	3.27%	1.09%	39.11%
B3	10199924	3968744	104273	13909	10859	87033	295361	937	220353	100324	501140	228401	4668590
B3 (percent)	100.00%	38.91%	1.02%	0.14%	0.11%	0.85%	2.90%	0.01%	2.16%	0.98%	4.91%	2.24%	45.77%
B8	13073698	6831682	31149	26799	8817	107581	365228	776	171375	40742	200158	80529	5217862

B8 (percent)	100.00%	52.26%	0.24%	0.20%	0.07%	0.82%	2.72%	0.01%	1.31%	1.53%	0.62%	0.62%	39.91%
C3	11886685	5583766	40945	161103	6661	127456	310086	820	273182	39880	204752	87903	5050031
C3 (percent)	100.00%	46.97%	0.34%	1.36%	0.06%	1.07%	2.61%	0.01%	2.30%	0.34%	1.72%	0.74%	42.48%
C7	10265833	3720987	30659	156596	8880	155388	1094471	516	526740	24591	241638	53017	4252350
C7 (percent)	100.00%	36.25%	0.30%	1.53%	0.09%	1.51%	10.66%	0.01%	5.13%	0.24%	2.35%	0.52%	41.42%
C10	11648895	6122783	31181	17684	4081	130417	168951	653	142429	30510	164445	63332	4772429
C10	100.00%	52.56%	0.27%	0.15%	0.04%	1.12%	1.45%	0.01%	1.22%	0.26%	1.41%	0.54%	40.97%
D1	10092981	5825375	19487	28060	3637	54518	85816	648	113869	14531	89757	33016	3824267
D1 (percent)	100.00%	57.72%	0.19%	0.28%	0.04%	0.54%	0.85%	0.01%	1.13%	0.14%	0.89%	0.33%	37.89%
D2	10980277	5506507	554114	19839	5940	123810	295301	868	168381	59717	309541	132925	4303334
D2 (percent)	100.00%	50.15%	0.49%	0.18%	0.05%	1.13%	2.69%	0.01%	1.53%	0.54%	2.82%	1.21%	39.19%
D3	13899690	7263692	39643	67429	5784	104998	296517	878	178336	32047	201774	62058	5646534
D3 (percent)	100.00%	52.26%	0.29%	0.49%	0.04%	0.76%	2.13%	0.01%	1.28%	0.23%	1.45%	0.45%	40.62%
E1	9807608	6212784	10621	3238	1636	52028	72301	369	27990	11631	70721	56473	3277816

E1 (percent)	100.00%	63.35%	0.11%	0.03%	0.02%	0.53%	0.74%	0.00%	0.39%	0.12%	0.72%	0.58%	33.42%
E6	9661029	5123747	28545	75351	6327	75574	160480	771	171946	21427	141435	39386	3816040
E6 (percent)	100.00%	53.04%	0.30%	0.78%	0.07%	0.78%	1.66%	0.01%	1.78%	0.22%	1.46%	0.41%	39.50%
E9	10171968	5512522	38809	11289	3897	60299	138366	832	121393	47183	210237	99861	39272279
E9 (percent)	100.00%	54.19%	0.38%	0.11%	0.04%	0.59%	1.36%	0.01%	1.19%	0.46%	2.07%	0.98%	38.61%
F5	9263494	4637459	24823	55995	6476	106484	225660	718	250534	19753	139438	47125	3749029
F5 (percent)	100.00%	50.06%	0.27%	0.60%	0.07%	1.15%	2.44%	0.01%	2.70%	0.21%	1.51%	0.51%	40.47%
F7	11498360	4386525	85307	250368	15708	108376	580561	573	283608	38234	351264	79459	5318377
F7 (percent)	100.00%	38.15%	0.74%	2.18%	0.14%	0.94%	5.05%	0.00%	2.47%	0.33%	3.05%	0.69%	46.25%
F10	10878172	6196044	18027	12468	7503	126535	302076	591	131528	39780	121884	47831	3873905
F10 (percent)	100.00%	56.96%	0.17%	0.11%	0,.07%	1.16%	2.78%	0.01%	1.21%	0.37%	1.12%	0.44%	35.61%
G1	9543915	4432083	43341	66308	13539	101871	287886	720	361246	45208	245434	94028	3852251
G1 (percent)	100.00%	46.44%	0.45%	0.69%	0.14%	1.07%	3.02%	0.01%	3,79%	0.47%	2.57%	0.99%	40.36%

G2	10179499	4347371	45119	45119	13713	129654	339322	785	472886	49994	307474	112198	4307953
G2 (percent)	100.00%	42.71%	0.44%	0.52%	0.13%	1.27%	3.33%	0.01%	4.65%	0.49%	3.02%	1.10%	43.32%
G8	9000777	4298224	17874	4828	6959	89223	1043608	367	94949	32965	200393	53731	3157656
G8 (percent)	100.00%	47.75%	0.20%	0.05%	0.08%	0.99%	11.59%	0.00%	1.05%	0.37%	2.23%	0.60%	35.08%
H1	11407697	5510667	29688	39334	6166	91689	173302	756	201718	27832	161974	69701	5094870
H1 (percent)	100.00%	48.31%	0.26%	0.34%	0.05%	0.80%	1.52%	0.01%	1.77%	0.24%	1.42%	0.61%	44.66%
H4	11935831	4615265	101132	52811	17705	146698	419309	757	536660	116568	612353	276510	5040063
H4 (percent)	100.00%	38.67%	0.85%	0.44%	0.15%	1.23%	3.51%	0.01%	4.50%	0.98%	5.13%	2.32%	42.23%
H9	11588383	5650623	57906	30250	9585	134558	211005	851	259547	56155	303262	134116	4740525
H9 (percent)	100.00%	48.76%	0.50%	0.26%	0.08%	1.16%	1.82%	0.01%	2.24%	0.48%	2.62%	1.16%	40.91%
I2	10609054	4309058	22657	67631	10883	121601	449848	570	426596	20348	222883	49493	4907486
I2 (percent)	100.00%	40.62%	0.21%	0.64%	0.10%	1.15%	4.24%	0.01%	4.02%	0.19%	2.10%	0.47%	46,26%
I4	12804597	6454381	33439	54707	9917	101670	201840	875	209406	34731	208570	74042	5421092
I4 (percent)	100.00%	50.41%	0.26%	0.43%	0.08%	0.79%	1.58%	0.01%	1.64%	0.27%	1.63%	0.58%	42.34%

I7	11548265	4851303	44504	57796	7554	85585	334233	792	229085	45044	285772	106891	5499706
I7 (percent)	100.00%	42.01%	0.39%	0.50%	0.07%	0.74%	2.89%	0.01%	1.98%	0.39%	2.47%	0.93%	47.62%
J2	10803163	4265750	82987	16619	11236	114573	401294	1087	233455	93272	445349	197157	4940384
J2 (percent)	100.00%	39.49%	0.77%	0.15%	0.10%	1.06%	3.71%	0.01%	2.16%	0.86%	4.12%	1.82%	45.73%
J6	10483749	4854940	54041	23719	5666	117066	270253	825	137414	43309	248659	95348	4632509
J6 (percent)	100.00%	46.31%	0.52%	0.23%	0.05%	1.12%	2.58%	0.01%	1.31%	0.41%	2.37%	0.91%	44.19%
J7	1266194	5524136	87610	12684	8269	126937	288270	984	180639	88913	443552	190770	5709180
J7 (percent)	100.00%	43.63%	0.69%	0.10%	0.07%	1.00%	2.28%	0.01%	1.43%	0.70%	3.50%	1.51%	45.09%
K1	11742813	5813863	44646	64883	8983	134745	282638	794	208819	50835	264058	105690	4762859
K1 (percent)	100.00%	49.51%	0.38%	0.55%	0.08%	1.15%	2.41%	0.01%	1.78%	0.43%	2.25%	0.90%	40.56%
K5	9610236	4316483	55220	2764	4728	90957	269267	735	116912	57841	288958	120055	4286316
K5 (percent)	100.00%	44.92%	0.57%	0.03%	0.05%	0.95%	2.80%	0.01%	1.22%	0.60%	3.01%	1.25%	44.60%
K6	10509833	3267238	101037	1553	23438	147016	801247	426	246649	154164	1014171	214581	4538313

K6 (percent)	100.00%	31.09%	0.96%	0.01%	0.22%	1.40%	7.62%	0.00%	2.35%	1.47%	9,65%	2.04%	43.18%
L1	11549158	4742850	38524	232359	24562	127099	532106	699	632988	32881	302033	66349	4810408
L1 (percent)	100.00%	41.07%	0.33%	2.01%	0.21%	1.10%	4.61%	0.01%	5.54%	0.28%	2.62%	0.57%	41.65%
L9	10360595	4647723	42609	53178	16186	104978	388417	830	415239	52345	341389	107633	4190068
L9 (percent)	100.00%	44.86%	0.41%	0.51%	0.16%	1.01%	3.75%	0.01%	4.01%	0.51%	3.30%	1.04%	40.44%
L10	10925397	3975475	108856	85895	14080	162498	439963	797	413565	123115	620475	241249	4739429
L10 (percent)	100.00%	36.39%	1.00%	0.79%	0.13%	1.49%	4.03%	0.01%	3.79%	1.13%	5.68%	2.21%	43.38%

Note：> total：Refers to the number of sRNAs in each sample compared to the reference sequence, followed by a reference to calculate the proportion of each type of sRNA.

> known_miRNA：Refers to the number and proportion of sRNAs that are compared to known miRNAs for each sample.

> rRNA/tRNA/snRNA/snoRNA：Refers to the number and proportion of rRNA/tRNA/snRNA/snoRNA sRNA in each sample.

> novel_miRNA：Refers to the number and proportion of sRNA in each sample compared to the new miRNA.

> exon：+/exon：-/intron：+/intron：-：Refers to a sample comparison to the number of exon/intron positive and negative chains and their proportion.

> other：Refers to the comparison of each sample to the reference sequence, but does not compare the number and proportion of sRNAs to known miRNAs, ncRNAs, new miRNAs, and exons and intron regions

Supplementary Table 7. 135 (partially repeated) differentially expressed miRNA expression levels between groups

miRNA	CON	MOD	PSF.L	PSF.M	PSF.H	PS.L	PS.M	PS.H	WE.L	WE.M	WE.H	SIM
rno-miR-200a-5p	81.91	276.75	620.04	400.67	386.67	495.67	462.00	270.00	390.00	314.67	372.33	401.00
rno-miR-3548	68.81	224.32	500.89	312.67	310.33	398.67	360.00	205.00	312.67	256.67	282.33	324.33
rno-miR-455-3p	99.07	200.60	184.67	319.00	187.67	256.67	204.33	187.33	193.33	277.00	418.00	157.00
rno-miR-155-5p	76.38	124.64	166.33	105.07	100.00	96.00	94.00	105.33	139.67	145.00	277.82	112.33
rno-miR-139-5p	1158.96	1701.24	2105.00	1955.33	1683.33	2347.00	2039.00	1989.67	2264.00	2525.67	3363.00	1916.00
rno-miR-200b-5p	12.68	33.93	41.00	50.33	25.33	42.00	39.67	25.33	25.33	31.67	64.67	35.67
rno-let-7g-3p	0.79	6.48	2.67	4.00	4.00	3.33	2.67	2.67	3.67	2.67	2.33	4.33
rno-miR-184	28.41	77.23	81.00	193.67	58.00	146.00	46.00	88.00	57.67	9.29	37.67	47.00
rno-miR-375-3p	1227.95	2132.58	3181.00	3307.33	3012.00	3838.00	2546.00	2396.33	2438.00	3168.67	2622.00	3351.33
rno-miR-126b	97.58	59.45	76.67	65.00	72.67	76.33	52.33	64.67	70.67	86.00	53.33	77.00
rno-miR-101a-3p	33591.11	22931.01	27996.33	31088.00	31374.67	30197.00	18560.67	27828.67	26014.00	29140.67	23893.33	33227.67
rno-miR-455-5p	876.80	1547.60	2488.67	1886.33	1693.67	1980.33	1591.33	2314.67	2666.67	2460.33	1146.33	1534.00

rno-miR-204-5p	35.71	12.79	48.67	42.00	41.67	32.67	24.67	42.33	43.67	42.33	36.00	47.33
rno-miR-144-5p	231.32	122.39	252.33	184.33	222.33	429.00	257.33	212.33	153.00	246.00	194.33	134.00
rno-miR-451-5p	1689.67	1608.98	5138.57	2480.33	2106.33	7869.33	6667.33	3802.33	2646.33	3025.33	3362.33	2310.67
rno-miR-27a-5p	148.33	129.96	355.80	164.00	165.00	148.00	172.00	180.67	249.67	258.33	221.00	110.00
rno-miR-486	644.67	731.48	1866.79	1192.33	1665.33	6529.67	3689.33	979.00	766.33	1087.33	1600.67	911.67
rno-miR-193b-3p	85.00	150.68	81.38	175.33	125.33	150.33	93.00	136.00	143.33	233.33	175.33	176.67
rno-miR-218a-5p	235.00	257.96	396.82	259.00	303.67	459.67	275.33	282.33	334.33	351.00	232.33	262.00
rno-miR-218b	220.00	241.68	369.71	241.67	288.00	436.33	260.67	265.67	318.33	334.00	218.00	249.00
rno-miR-6329	77.00	88.17	150.34	141.61	109.00	129.67	141.61	161.69	136.60	142.00	123.00	126.67
novel_380	96.33	152.08	69.97	113.67	86.00	68.81	85.00	110.33	73.96	168.00	166.33	137.33
rno-miR-674-5p	100.67	144.02	74.62	142.67	72.00	120.67	81.00	95.33	103.33	155.00	136.33	94.00
rno-miR-34a-5p	295.00	594.17	1008.10	1082.67	1037.31	1168.33	423.67	722.33	862.67	1037.31	663.33	884.00
rno-miR-27b-5p	30.67	33.11	63.66	64.67	46.00	43.67	42.00	56.00	55.67	62.25	51.33	45.67
rno-miR-872-3p	54.33	67.10	36.17	55.33	41.67	64.67	39.67	44.67	62.67	103.00	46.33	71.33

rno-miR-3591	570349.00	678984.32	441138.29	919571.00	458747.33	695366.67	404941.67	559518.33	444524.91	457994.27	640041.33	486635.00
rno-miR-194-5p	58675.33	77880.16	51100.88	75951.67	52896.33	60330.00	46669.00	64133.33	61899.00	84747.67	40791.33	66772.33
rno-miR-122-5p	924354.33	1045670.27	643091.25	1273570.33	630795.67	1078007.00	588732.67	837387.00	748935.00	842153.33	1007918.33	761266.33
rno-miR-1247-3p	31.67	24.10	52.61	74.28	47.00	22.00	21.67	28.00	45.33	33.67	113.67	15.33
rno-miR-146b-5p	540.33	683.49	1116.24	881.33	629.33	954.00	650.00	640.67	832.33	746.33	1085.33	612.00
rno-miR-3556b	396.00	433.77	260.74	410.33	305.67	385.67	278.33	373.00	417.00	643.00	246.33	474.33
rno-miR-29c-3p	410.33	447.12	269.89	423.33	312.00	396.67	285.67	388.00	427.67	659.00	250.00	489.33
rno-miR-21-3p	54.67	54.94	64.00	13.21	29.67	32.67	20.82	34.33	39.33	46.67	37.33	40.00
rno-miR-142-3p	387.33	697.61	884.67	256.23	322.05	840.67	441.67	713.00	840.67	1160.67	968.33	668.33
rno-miR-32-5p	178.67	226.89	257.67	114.40	144.00	217.33	124.00	202.00	240.00	329.33	176.67	238.00
rno-miR-19a-3p	54.00	62.52	86.67	18.37	22.67	58.00	29.67	62.67	70.33	89.00	32.00	65.33
rno-miR-130a-3p	76.33	94.25	90.67	51.27	67.33	85.00	61.67	76.67	88.33	87.67	77.33	104.33
rno-miR-3590-5p	76.33	94.25	90.67	51.27	67.33	85.00	61.67	76.67	88.33	87.67	77.33	104.33

rno-miR-203b-5p	2798.67	3085.61	3362.67	4594.62	3752.67	3249.33	2058.67	3388.67	3043.33	3056.00	1764.00	2892.33
rno-miR-3065-5p	22.00	29.81	27.00	15.06	17.33	28.67	12.33	27.33	37.67	38.00	19.33	22.33
rno-miR-338-3p	22.00	29.81	27.00	15.06	17.33	28.67	12.33	27.33	37.67	38.00	19.33	22.33
rno-miR-1843a-5p	974.67	1044.50	1196.00	1512.91	1341.33	1174.67	918.33	1392.00	1495.00	1579.00	1235.00	1222.00
rno-miR-107-3p	690.67	868.80	676.67	617.08	578.33	791.33	498.00	778.00	814.67	1045.67	606.67	877.33
rno-miR-203a-3p	4105.33	4763.84	5281.33	6758.27	5787.67	4946.33	3118.00	5487.67	4847.67	4721.67	2681.33	4344.00
rno-miR-144-3p	137.00	116.69	222.67	59.58	134.67	377.67	139.33	157.00	155.00	316.45	219.33	183.67
rno-miR-181a-1-3p	75.33	105.57	158.33	170.73	142.67	117.00	91.33	130.67	111.00	119.33	118.33	105.00
rno-miR-3570	75.33	105.57	158.33	170.73	142.67	117.00	91.33	130.67	111.00	119.33	118.33	105.00
rno-miR-181a-2-3p	13.33	8.94	11.67	20.01	10.00	7.00	11.67	15.33	15.00	11.67	15.67	9.67
rno-miR-3120	30.67	48.06	53.67	26.48	33.67	61.00	42.33	51.00	43.33	55.67	64.33	51.67
rno-miR-19b-3p	761.00	995.54	802.67	539.22	497.33	997.67	527.67	852.00	715.33	1114.33	453.00	923.67
rno-miR-653-5p	1.33	0.00	1.67	2.81	0.67	1.33	1.00	1.00	1.33	0.67	0.67	0.33

rno-miR-362-3p	113.00	131.72	102.67	78.52	71.67	113.33	54.64	95.33	85.33	159.67	67.00	135.67
rno-miR-186-5p	1673.33	2332.57	1910.00	1567.44	1136.86	2664.33	1186.67	1822.67	2018.67	2830.67	1620.00	2258.67
rno-miR-15a-3p	0.33	2.43	0.00	0.00	0.33	0.33	0.33	0.67	0.67	0.00	0.67	0.67
rno-miR-33-5p	22.33	17.75	7.67	5.61	7.00	9.67	4.33	12.00	19.00	20.00	7.67	13.67
rno-miR-1843b-5p	169.00	176.26	192.67	240.11	208.67	206.00	158.33	226.67	237.67	282.33	222.33	214.00
rno-miR-378b	8.33	11.23	15.67	111.33	199.71	101.00	243.52	13.00	17.00	18.33	23.67	14.00
rno-miR-542-3p	909.67	799.28	1376.00	1201.67	1597.65	1028.00	846.00	1135.67	1109.33	1209.33	1076.67	1229.67
rno-miR-203a-5p	637.33	648.07	886.33	1303.67	1465.35	783.00	572.67	839.33	918.67	792.00	526.33	952.67
novel_536	49.33	114.89	21.33	46.67	30.02	28.00	43.33	80.00	39.33	124.33	33.33	50.67
rno-miR-203b-3p	825.33	838.41	1150.33	1635.67	1740.82	989.67	705.00	1096.00	1210.67	1026.67	681.67	1222.00
rno-miR-421-3p	5.00	2.81	5.67	4.33	5.67	12.86	5.00	5.00	5.00	6.67	8.33	7.00
rno-miR-93-3p	5.33	3.94	9.67	11.67	8.33	15.40	6.67	6.00	7.33	8.67	10.67	6.67
rno-miR-98-3p	2.00	3.50	2.00	1.67	1.67	0.34	1.33	2.00	1.67	2.00	2.33	4.15
rno-miR-106b-3p	192.67	248.53	354.33	450.33	367.67	532.00	499.01	332.33	329.33	359.33	556.00	328.33

rno-miR-490-3p	18.33	20.60	13.33	21.67	25.00	23.00	8.74	13.67	22.33	27.67	20.67	20.67
rno-miR-96-5p	203.33	238.69	148.67	188.67	166.67	335.67	102.21	152.00	446.33	289.33	277.33	149.67
rno-miR-429	188.33	339.16	441.33	338.00	419.00	535.00	336.33	236.61	393.67	467.33	352.33	504.33
rno-miR-148a-5p	4487.00	6030.83	8861.67	8273.00	7145.67	7050.67	6889.00	9719.31	11180.73	10375.67	6962.00	7855.67
rno-miR-31a-3p	116.00	109.54	127.33	172.67	176.33	156.67	109.67	178.65	174.00	170.33	113.00	174.33
rno-miR-217-5p	15.33	13.29	29.33	12.33	23.00	124.00	23.33	4.47	6.00	13.33	19.00	14.00
rno-miR-183-5p	276.67	314.03	213.67	403.00	384.33	407.67	188.00	267.00	575.28	388.00	539.33	233.33
rno-miR-3553	276.67	314.03	213.67	403.00	384.33	407.67	188.00	267.00	575.28	388.00	539.33	233.33
rno-miR-7b	14.33	19.00	26.00	27.33	24.67	27.67	21.00	31.33	40.86	32.00	33.33	20.67
rno-miR-98-5p	328.00	406.49	390.33	423.33	307.00	602.00	340.67	355.33	282.21	400.67	285.00	323.00
rno-miR-182	1095.33	1186.15	810.00	1458.67	1487.33	1220.33	709.33	970.00	1722.14	1135.00	1464.67	892.00
rno-miR-200c-3p	76.33	103.43	112.33	89.33	55.33	154.33	56.00	70.00	70.33	55.17	87.00	51.08
rno-miR-379-5p	10.33	15.73	21.33	9.33	8.33	26.00	4.00	14.33	13.67	4.17	7.67	6.67
rno-miR-200b-3p	2283.67	5447.73	5296.33	5120.33	3902.33	5683.67	4797.33	3779.67	3540.67	3445.98	3953.33	4611.33

rno-miR-411-5p	5.33	4.83	6.33	3.33	2.00	4.33	2.67	5.67	3.67	0.83	2.00	1.33
rno-miR-6216	21.33	38.28	48.33	37.67	31.00	40.67	22.67	32.67	32.00	15.82	68.67	23.67
rno-miR-484	7.67	4.17	7.00	11.33	7.33	11.00	8.33	8.00	9.00	12.94	15.56	9.67
rno-miR-1b	784.33	896.10	1266.33	1897.33	1142.00	2594.67	3693.67	488.00	604.00	283.46	1011.67	276.67
rno-miR-127-3p	37.00	50.89	76.33	56.67	45.67	94.67	31.67	57.00	60.00	26.94	72.33	27.33
rno-miR-92b-3p	2.00	10.22	3.00	3.00	3.33	4.67	3.33	4.00	4.33	5.00	10.22	2.00
rno-miR-138-1-3p	1.33	3.03	1.00	1.33	1.33	1.33	0.33	0.00	1.33	0.33	3.03	3.33
rno-miR-3574	313.33	216.37	322.67	365.67	259.00	372.33	302.33	362.33	325.67	603.67	216.37	361.67
rno-miR-342-5p	4.33	9.22	6.67	2.00	2.67	3.67	3.00	3.33	2.00	2.33	9.22	3.33
rno-miR-210-3p	314.00	217.09	322.67	366.00	259.00	372.67	303.33	362.33	326.67	604.67	217.09	361.67
rno-miR-99a-3p	47.67	31.19	50.33	81.33	63.33	60.00	45.00	79.67	74.67	81.00	31.19	54.67
rno-miR-138-1-3p	1.33	0.00	1.00	1.33	1.33	1.33	0.33	0.00	1.33	0.33	3.00	3.27
rno-miR-150-3p	4.67	14.07	8.33	11.00	8.33	7.33	6.33	11.67	8.67	13.67	17.67	5.80

rno-miR-1843a-3p	32.00	37.48	48.00	61.00	39.67	57.67	34.67	46.33	46.00	71.67	85.00	59.89
rno-miR-582-5p	5.67	10.70	11.00	8.67	5.67	8.67	8.33	6.33	6.33	9.33	4.00	4.15
rno-miR-547-5p	1.33	2.49	0.67	2.00	0.00	1.67	1.00	2.00	0.67	1.00	2.00	0.00
rno-miR-122-3p	2113.33	2676.75	3290.33	3446.33	3122.67	2985.00	2737.67	3226.00	3063.00	3416.00	2797.67	3700.92

Supplementary Table 8. Groups of 11 miRNAs that are significantly different from the MOD group

miRNA	TEST
miR-19a-3p	PSF.M
miR-21-3p	PSF.M
miR-142-3p	PSF.M
miR-1247-3p	PSF.M
miR-200a-5p	CON
miR-3548	CON
miR-122-5p	PSF.L
miR-451-5p	PSF.L
miR-378b	PS.M
miR-184	PWE.M
miR-484	PWE.H

Supplementary Table 9. Three software predicts the correspondence between the analyzed miRNA and the target gene (partial).

Target::miRNA	RNAhybrid	PITA	miRanda
ENSRNOT00000000008::rno-miR-101a-3p	0	0	1
ENSRNOT00000000008::rno-miR-124-5p	0	0	1
ENSRNOT00000000008::rno-miR-150-3p	1	0	0
ENSRNOT00000000008::rno-miR-188-5p	1	1	0
ENSRNOT00000000008::rno-miR-350	0	1	1
ENSRNOT00000000008::rno-miR-3541	0	1	0
ENSRNOT00000000008::rno-miR-674-5p	1	1	0
ENSRNOT00000000008::rno-miR-872-3p	0	1	0
ENSRNOT00000000009::novel_392	1	0	0
ENSRNOT00000000010::novel_549	1	0	0
ENSRNOT00000000008::novel_150	0	0	1
ENSRNOT00000000008::novel_183	0	1	0
ENSRNOT00000000008::novel_239	0	1	0
ENSRNOT00000000008::novel_226	1	1	0

Note：0 means unpredicted, 1 means predicted.

Supplementary Table 10. Gene Ontology Enrichment List of Candidate Target Genes in Samples (Partial)

GO_accession	Description	P-value
GO:0044241	lipid digestion	0.000894620
GO:0036146	cellular response to mycotoxin	0.000018207
GO:0009137	purine nucleoside diphosphate catabolic process	0.000025044
GO:0009191	ribonucleoside diphosphate catabolic process	0.000062470
GO:0009155	purine deoxyribonucleotide catabolic process	0.000062470
GO:0009181	purine ribonucleoside diphosphate catabolic process	0.000062119
GO:0009134	nucleoside diphosphate catabolic process	0.000087353
GO:0009151	purine deoxyribonucleotide metabolic process	0.000186490
GO:0046386	deoxyribose phosphate catabolic process	0.000227640
GO:0009264	deoxyribonucleotide catabolic process	0.000272820
GO:0016077	snoRNA catabolic process	0.002071700
GO:0072237	metanephric proximal tubule development	0.006202600
GO:0006195	purine nucleotide catabolic process	0.006457100
GO:0060548	negative regulation of cell death	0.006619600
GO:0034314	Arp2/3 complex-mediated actin nucleation	0.007808700
GO:0060587	regulation of lipoprotein lipid oxidation	0.008186600
GO:0034439	lipoprotein lipid oxidation	0.008186600
GO:0001635	calcitonin gene-related peptide receptor activity	0.008231000
GO:1902463	protein localization to cell leading edge	0.008564900
GO:2001025	positive regulation of response to drug	0.008564900

GO:0015670	carbon dioxide transport	0.010317000
GO:0043029	T cell homeostasis	0.015289000
GO:0090130	tissue migration	0.040841000

Supplementary Table 11. Regulation of Intestinal Microbial Composition of Rats with Abnormal Lipid Metabolism (abundance)

	CON	MOD	PSF.L	PSF.M	PSF.H	PS.L	PS.M	PS.H	WE.L	WE.M	WE.H	SIM
Alloprevotella	0.24±0.00	0.12±0.00	0.12±0.00	0.21±0.00	0.06±0.00	0.07±0.00	0.05±0.00	0.07±0.00	0.44±0.00**	0.26±0.00	0.18±0.00	0.14±0.00
Allobaculum	0.71±0.00	0.53±0.00	0.59±0.00	0.44±0.00	0.39±0.00	0.64±0.00	0.83±0.01	0.79±0.00	0.57±0.00	0.64±0.00	0.84±0.00*	0.75±0.00
Bacteroides	2.65±0.02	2.34±0.01	2.12±0.01	6.65±0.03**	3.35±0.00	3.42±0.01	2.59±0.01	3.10±0.01	2.76±0.01	2.76±0.01	5.61±0.02*	2.05±0.01
Blautia	0.77±0.00	0.62±0.00	0.87±0.00	1.27±0.00	0.73±0.00	1.23±0.01	1.40±0.01	1.52±0.00	0.67±0.00	1.12±0.00	2.08±0.00*	1.16±0.00
Bacillus	0.59±0.00	0.65±0.00	0.62±0.00	0.91±0.00	1.66±0.01	0.47±0.00	0.55±0.00	0.29±0.00	0.27±0.00	0.34±0.00	0.67±0.00	0.35±0.00
Clostridium sensu stricto_1	1.45±0.00	1.26±0.00	1.64±0.00	1.44±0.00	2.78±0.02	0.71±0.01	0.60±0.01	0.09±0.00	0.11±0.00	0.11±0.00	1.21±0.00	1.06±0.01
*Christensenellaceae_*– R-7	0.39±0.00*	0.61±0.00	0.46±0.00	0.42±0.00*	0.44±0.00	0.52±0.00	0.58±0.00	0.59±0.00	0.35±0.00*	0.45±0.00	0.29±0.00**	0.39±0.00*
Coprococcus_1	0.20±0.00	0.17±0.00	0.18±0.00	0.21±0.00	0.15±0.00	0.17±0.00	0.19±0.00	0.20±0.00	0.15±0.00	0.20±0.00	0.24±0.00	0.22±0.00
Desulfovibrio	0.40±0.00	0.62±0.00	0.59±0.00	0.79±0.00	0.85±0.00	0.95±0.01	0.71±0.00	0.67±0.00	0.72±0.00	1.01±0.00	0.57±0.00	0.53±0.00
[Eubacterium]_ruminantium	0.26±0.00	0.14±0.00	0.33±0.00	0.13±0.00	0.09±0.00	0.19±0.00	0.17±0.00	0.11±0.00	0.16±0.00	0.14±0.00	0.25±0.00	0.56±0.00*
[Eubacterium]_coprostanoligenes	0.57±0.00	0.81±0.00	0.73±0.00	0.74±0.00	0.79±0.00	0.69±0.00	0.91±0.00	0.77±0.00	0.55±0.00	0.60±0.00	0.79±0.00	0.82±0.00

Helicobacter	0.32±0.00	0.17±0.00	0.52±0.00*	0.31±0.00	0.42±0.00	0.24±0.00	0.37±0.00	0.30±0.00	0.44±0.00	0.54±0.00*	0.43±0.00	0.60±0.00*
Lactobacillus	11.54±0.02*	15.08±0.02	8.67±0.01**	8.72±0.01**	7.40±0.02***	12.08±0.03	9.77±0.03**	7.89±0.02***	5.60±0.03***	5.99±0.01***	8.25±0.01**	9.41±0.03**
*Lachnospiraceae*_NK4A136	4.97±0.01	4.05±0.01	5.26±0.01	3.70±0.00	4.44±0.01	4.49±0.02	3.72±0.01	2.59±0.00	3.26±0.01	4.19±0.02	3.03±0.00	5.13±0.01
Roseburia	1.41±0.00	1.02±0.00	1.76±0.01	2.56±0.01	1.90±0.01	1.66±0.00	1.79±0.00	1.95±0.01	1.83±0.01	4.25±0.00**	3.63±0.01*	2.37±0.00
*Ruminococcus*_1	2.79±0.01	2.73±0.00	2.82±0.00	2.09±0.01*	1.74±0.00**	2.24±0.01	2.29±0.00	2.16±0.00	1.94±0.01*	1.88±0.00*	1.69±0.01**	2.28±0.00
*Ruminococcaceae*_UCG-014	1.87±0.01	1.76±0.00	2.25±0.00	1.69±0.01	1.71±0.00	1.80±0.00	2.26±0.00	1.93±0.00	1.66±0.00	1.71±0.00	1.93±0.00	1.93±0.00
*Ruminiclostridium*_9	0.94±0.00	0.69±0.00	1.14±0.00	0.99±0.00	0.71±0.00	0.67±0.00	0.85±0.00	0.69±0.00	0.73±0.00	1.00±0.00	1.32±0.00	1.36±0.00
*Ruminococcaceae*_NK4A214	0.55±0.00	0.66±0.00	0.60±0.00	0.54±0.00	0.48±0.00	0.58±0.00	0.65±0.00	0.64±0.00	0.52±0.00	0.56±0.00	0.54±0.00	0.66±0.00
*Prevotella*_9	2.23±0.01	2.26±0.01	2.33±0.01	4.35±0.01	3.51±0.01	3.41±0.02	1.72±0.01	3.27±0.02	2.40±0.01	3.54±0.01	5.46±0.01*	2.65±0.02
*Prevotellaceae*_Ga6A1	1.98±0.00	1.73±0.01	1.41±0.01	1.58±0.01	1.40±0.01	1.66±0.01	0.65±0.00	1.64±0.01	1.37±0.00	0.69±0.00	1.53±0.00	1.32±0.01
Pseudoxanthomonas	0.13±0.00	0.23±0.00	0.18±0.00	0.15±0.00	1.21±0.01	0.12±0.00	0.07±0.00	0.02±0.00	0.04±0.00	0.01±0.00	0.14±0.00	0.13±0.00
Psychrobacter	0.25±0.00*	0.57±0.00	0.27±0.00*	0.26±0.00*	0.23±0.00**	0.38±0.00	0.31±0.00**	0.23±0.00**	0.39±0.00	0.22±0.00**	0.39±0.00	0.24±0.00**
Parabacteroides	0.20±0.00	0.22±0.00	0.24±0.00	0.33±0.00	0.26±0.00	0.42±0.00	0.29±0.00	0.36±0.00	0.28±0.00	0.29±0.00	0.30±0.00	0.23±0.00

Sutterella	0.16±0.00	0.26±0.00	0.19±0.00	0.24±0.00	0.23±0.00	0.36±0.00	0.41±0.00	0.39±0.00	0.25±0.00	0.33±0.00	0.30±0.00	0.20±0.00
Turicibacter	0.57±0.00	0.47±0.00	0.56±0.00	0.63±0.00	1.16±0.01	0.27±0.00	0.25±0.00	0.16±0.00	0.15±0.00	0.22±0.00	0.53±0.00	0.41±0.00
Terrisporobacter	0.83±0.00	0.74±0.00	0.96±0.00	0.84±0.00	1.74±0.01	0.41±0.00	0.35±0.01	0.01±0.00	0.01±0.00	0.01±0.00	0.78±0.00	0.60±0.01
Thermobacillus	0.23±0.00	0.15±0.00	0.16±0.00	0.29±0.00	0.31±0.00	0.07±0.00	0.05±0.00	0.00±0.00	0.00±0.00	0.01±0.00	0.23±0.00	0.08±0.00
Oscillibacter	0.52±0.00	0.46±0.00	0.77±0.00	0.71±0.00	0.66±0.00	0.59±0.00	0.76±0.00	0.64±0.00	0.96±0.01*	0.96±0.00*	0.79±0.00	0.76±0.00

Note: * P <0.05, ** P <0.01, *** P <0.001 compared with model group

1.2 Supplementary Figures

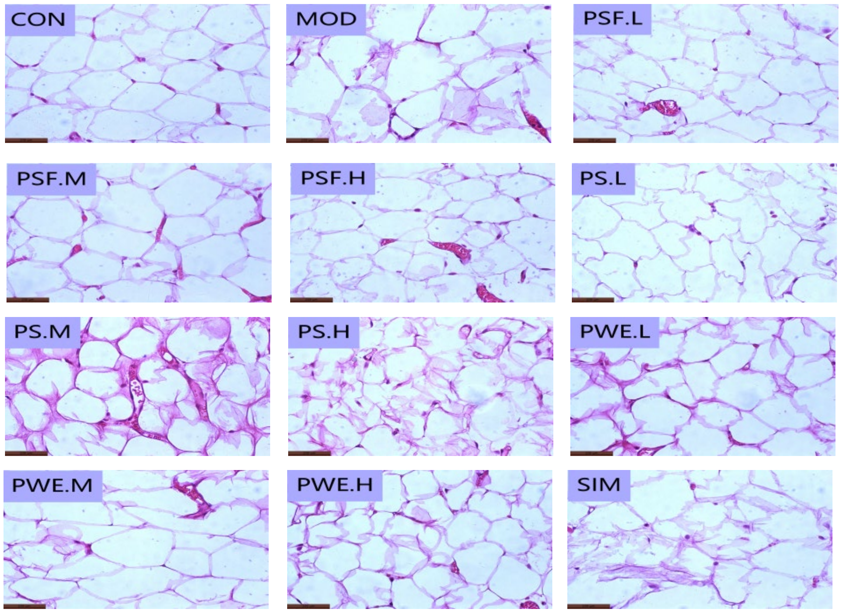

Supplementary Figure 1-1. HE staining of rat epididymal adipose tissue (×400)

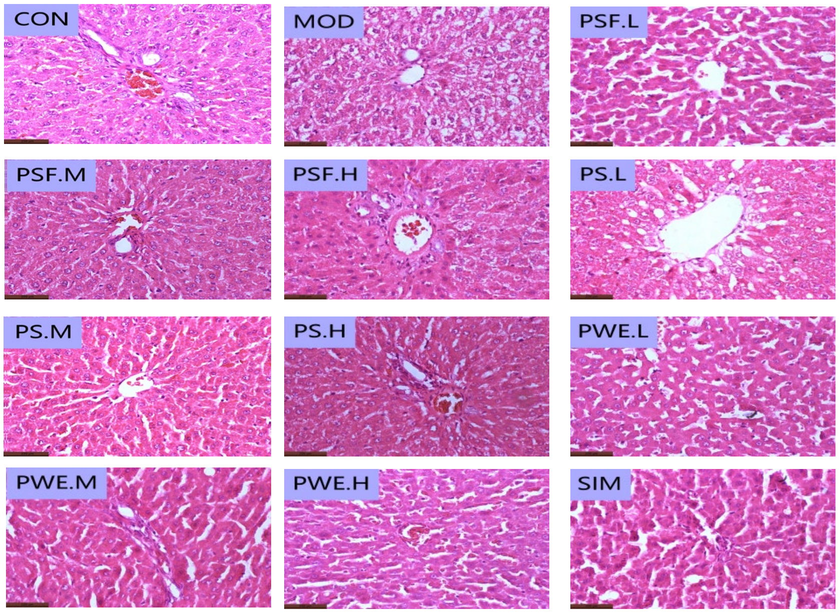

Supplementary Figure 1-2. HE staining of rat liver tissue (×200)

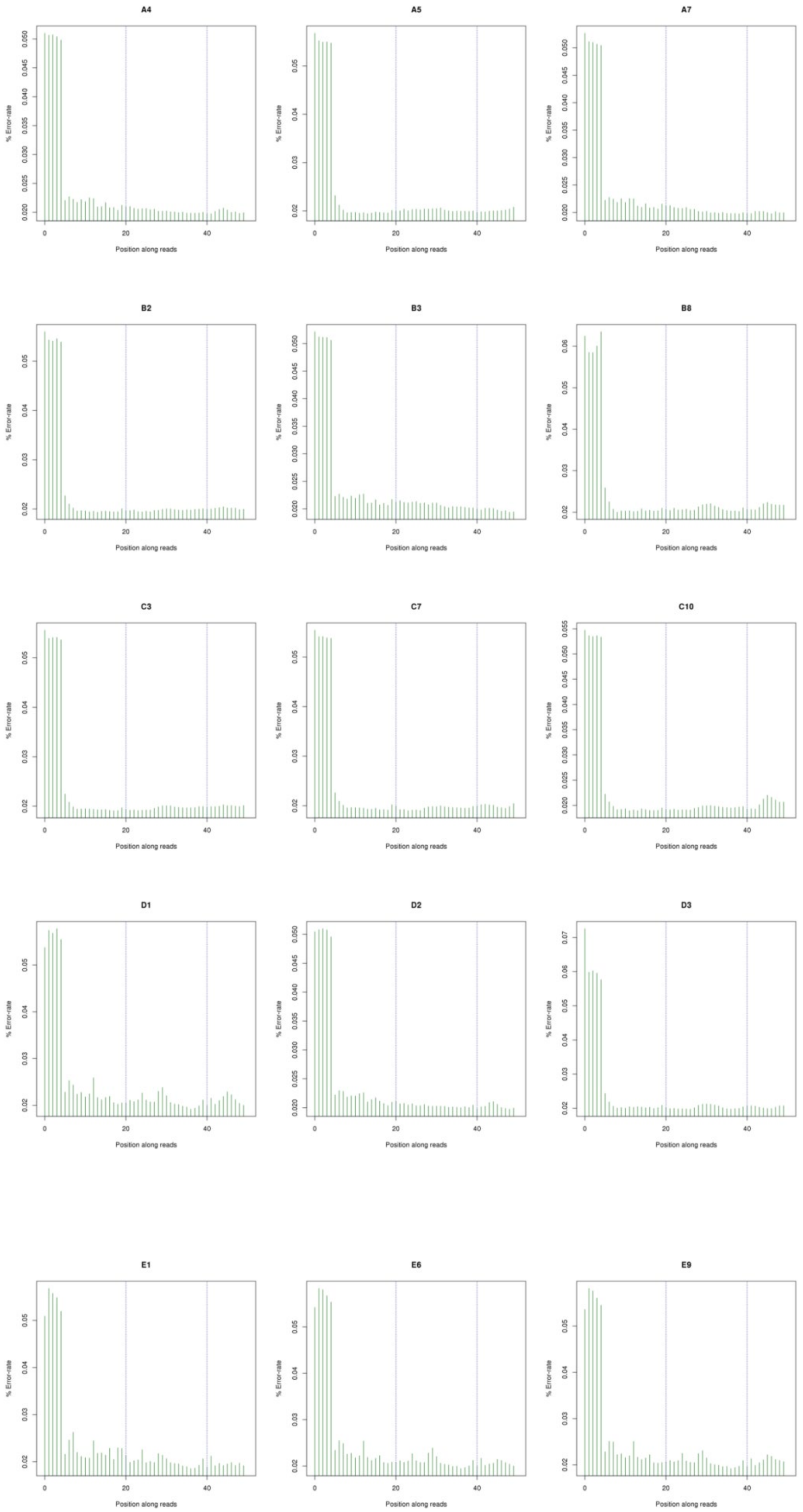
A4
A5
A7
B2
B3
B8
C3
C7
C10
D1
D2
D3
E1
E6
E9
% Error-rate
Position along reads

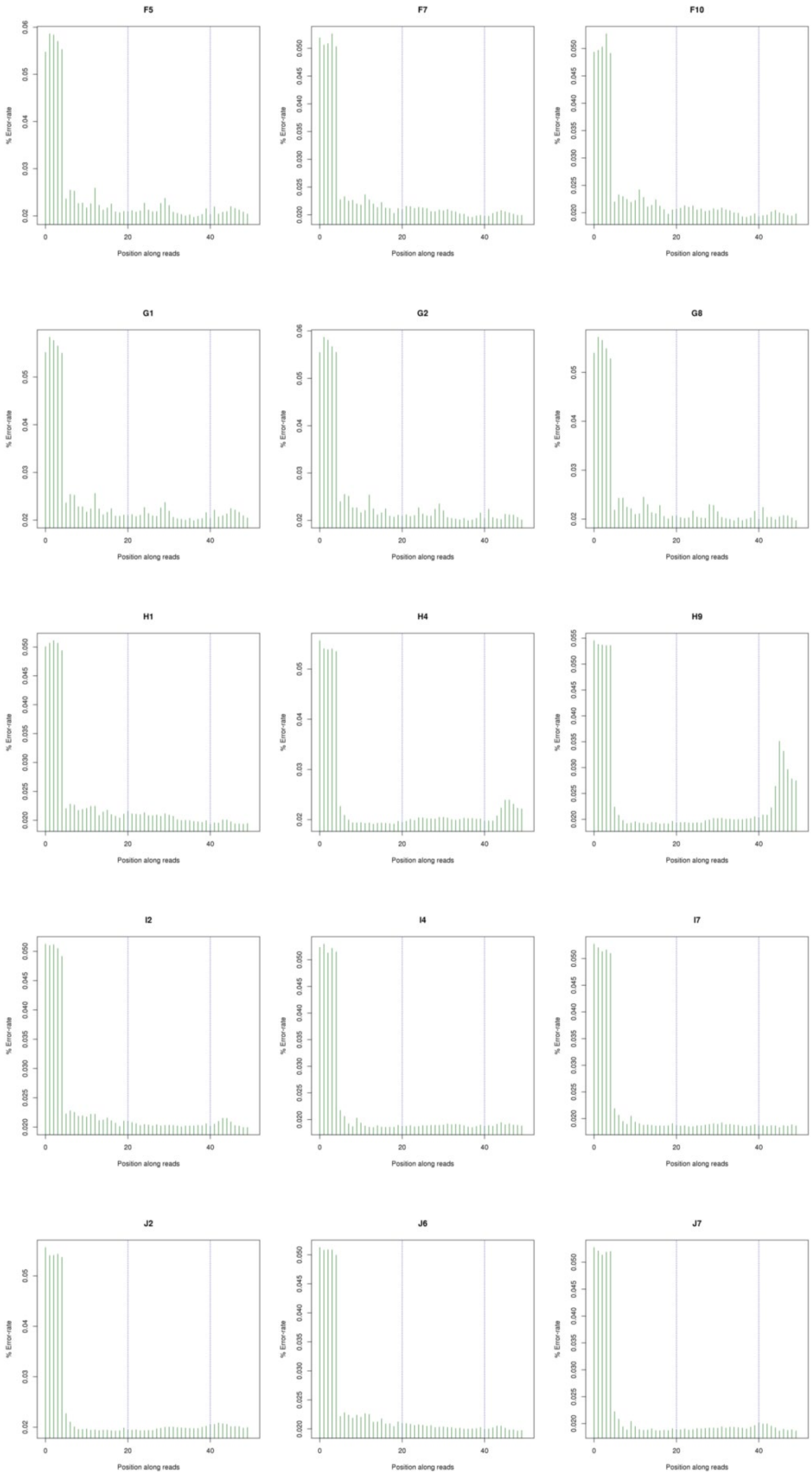
F5
F7
F10
G1
G2
G8
H1
H4
H9
I2
I4
I7
J2
J6
J7
% Error-rate
Position along reads

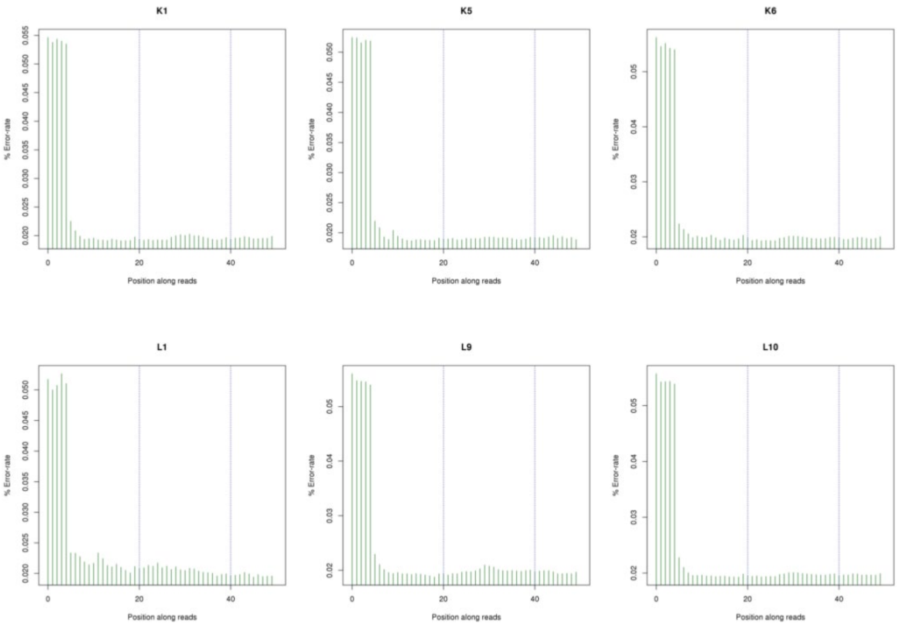

Supplementary Figure 2. Sequencing error rate distribution.

The abscissa is the base position of the reads, and the ordinate is the single base error rate

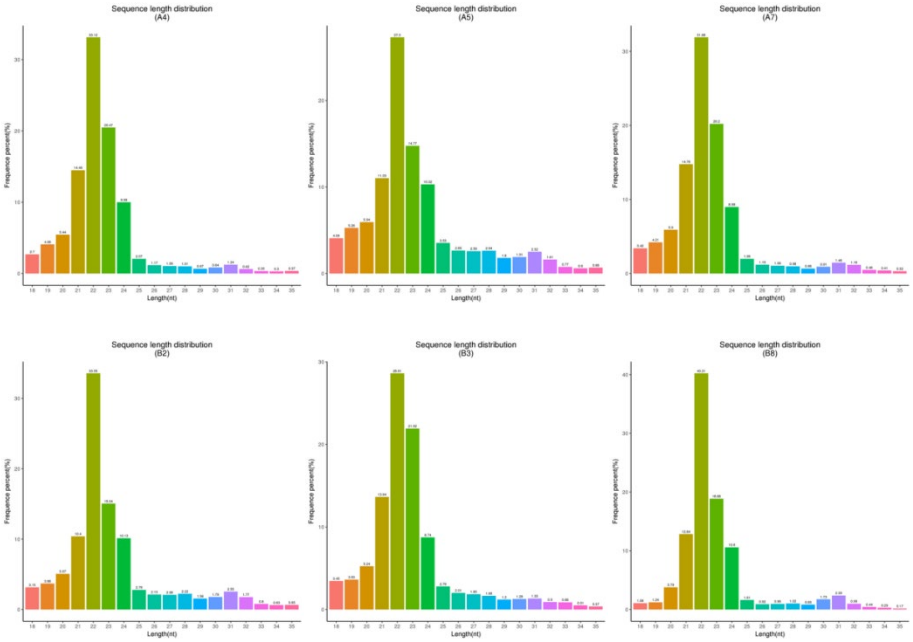

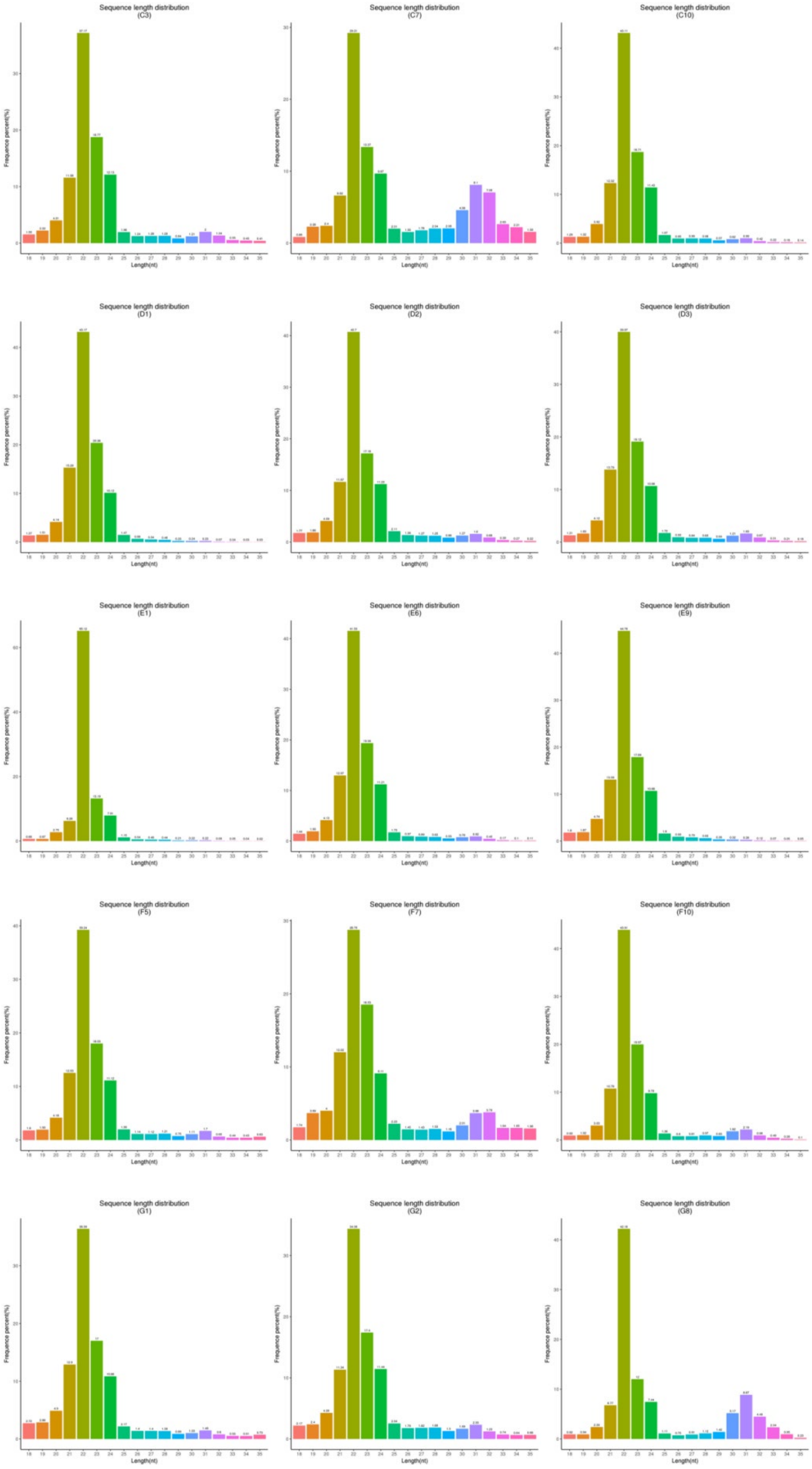
Sequence length distribution
(C3)
Sequence length distribution
(C7)
Sequence length distribution
(C10)
Sequence length distribution
(D1)
Sequence length distribution
(D2)
Sequence length distribution
(D3)
Sequence length distribution
(E1)
Sequence length distribution
(E6)
Sequence length distribution
(E9)
Sequence length distribution
(F5)
Sequence length distribution
(F7)
Sequence length distribution
(F10)
Sequence length distribution
(G1)
Sequence length distribution
(G2)
Sequence length distribution
(G8)
Frequence percent(%)
Length(nt)

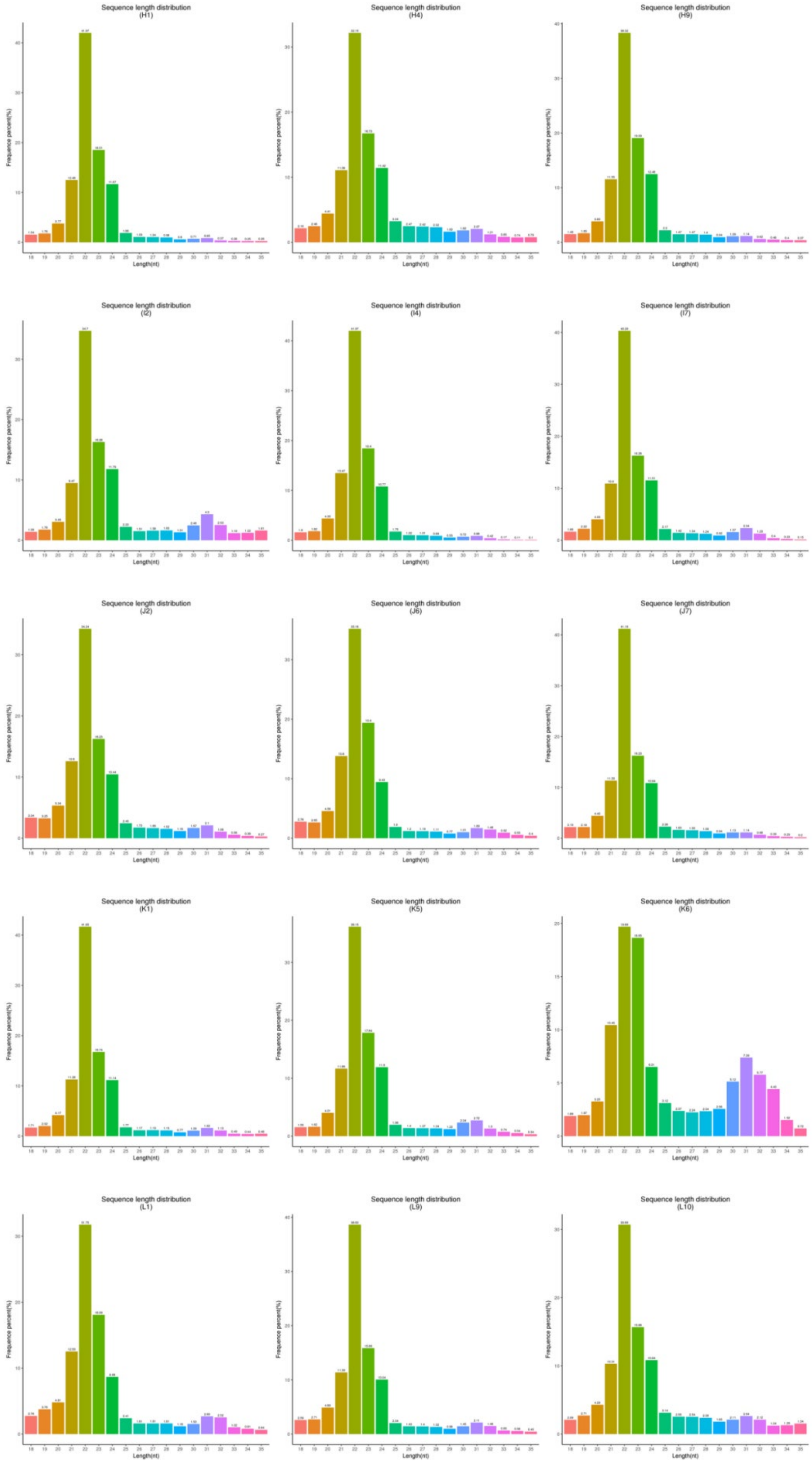

Supplementary Figure 3. Length distribution statistics of the obtained total sRNA fragments

The abscissa is the length of the reads, and the ordinate is the proportion of the reads of the length.

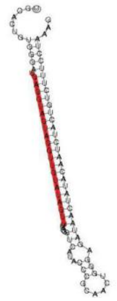

Supplementary Figure 4. Secondary structure of known miRNAs on partial matches

The entire sequence is the miRNA precursor, and the red highlight is the mature sequence.

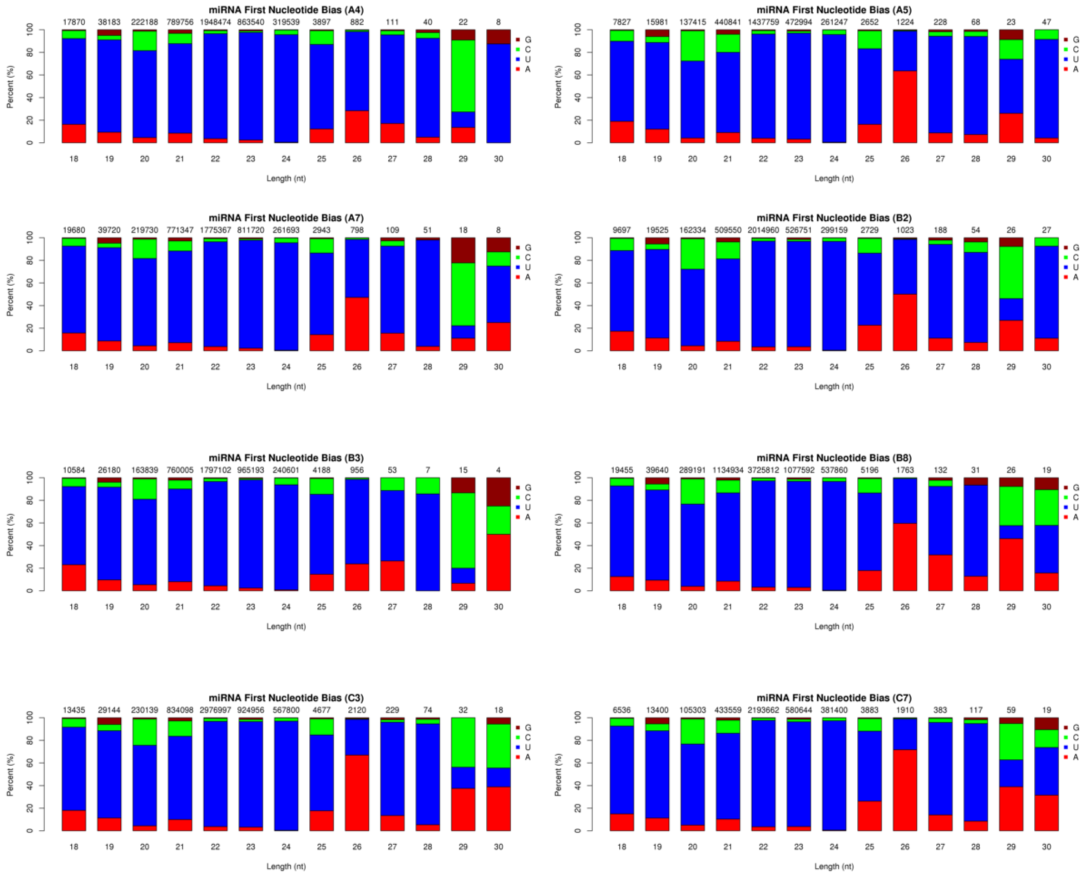

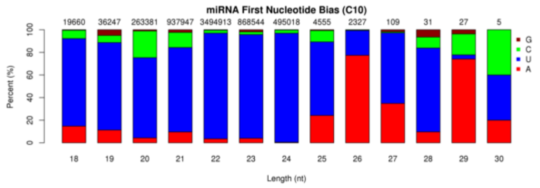
miRNA First Nucleotide Bias (C10)
19660 36247 263381 937947 3494913 868544 495018 4555 2327 109 31 27 5
Percent (%)
Length (nt)
G C U A

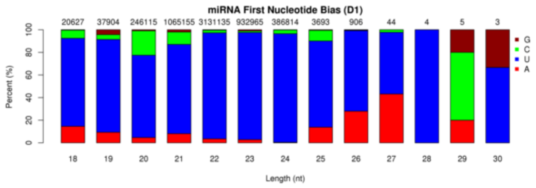
miRNA First Nucleotide Bias (D1)
20627 37904 246115 1065155 3131135 932965 386814 3693 906 44 4 5 3
Percent (%)
Length (nt)
G C U A

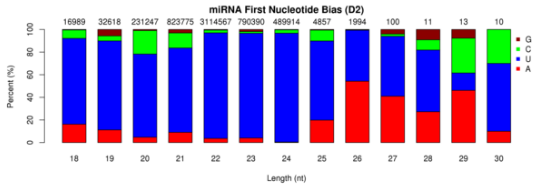
miRNA First Nucleotide Bias (D2)
16989 32618 231247 823775 3114567 790390 489914 4857 1994 100 11 13 10
Percent (%)
Length (nt)
G C U A

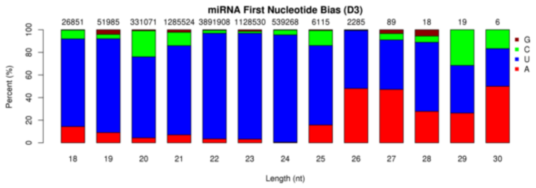
miRNA First Nucleotide Bias (D3)
26851 51985 331071 1285524 3891908 1128530 539268 6115 2285 89 18 19 6
Percent (%)
Length (nt)
G C U A

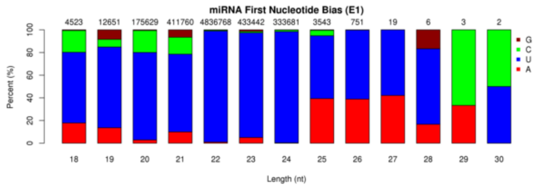
miRNA First Nucleotide Bias (E1)
4523 12651 175629 411760 4836768 433442 333681 3543 751 19 6 3 2
Percent (%)
Length (nt)
G C U A

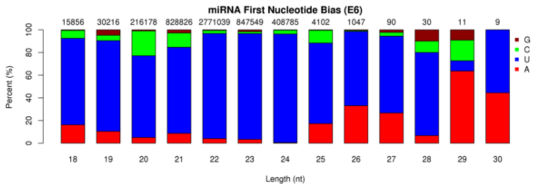
miRNA First Nucleotide Bias (E6)
15856 30216 216178 828826 2771039 847549 408785 4102 1047 90 30 11 9
Percent (%)
Length (nt)
G C U A

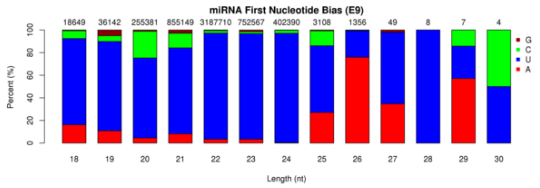
miRNA First Nucleotide Bias (E9)
18649 36142 255381 855149 3187710 752567 402390 3108 1356 49 8 7 4
Percent (%)
Length (nt)
G C U A

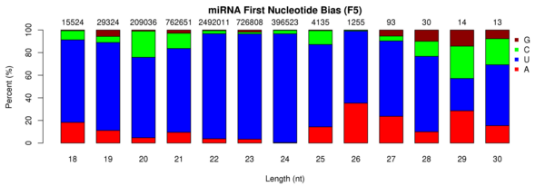
miRNA First Nucleotide Bias (F5)
15524 29324 209036 762651 2492011 726808 396523 4135 1255 93 30 14 13
Percent (%)
Length (nt)
G C U A

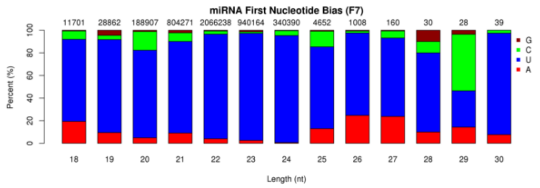
miRNA First Nucleotide Bias (F7)
11701 28862 188907 804271 2066238 940164 340390 4652 1008 160 30 28 39
Percent (%)
Length (nt)
G C U A

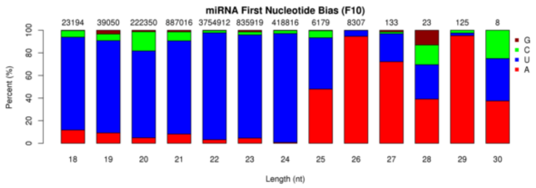
miRNA First Nucleotide Bias (F10)
23194 39050 222350 887016 3754912 835919 418816 6179 8307 133 23 125 8
Percent (%)
Length (nt)
G C U A

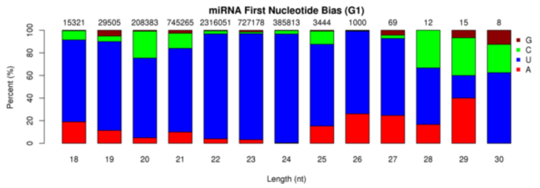
miRNA First Nucleotide Bias (G1)
15321 29505 208383 745265 2316051 727178 385813 3444 1000 69 12 15 8
Percent (%)
Length (nt)
G C U A

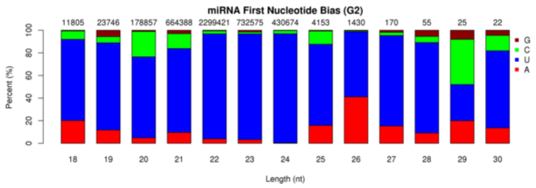
miRNA First Nucleotide Bias (G2)
11805 23746 178857 664388 2299421 732575 430674 4153 1430 170 55 25 22
Percent (%)
Length (nt)
G C U A

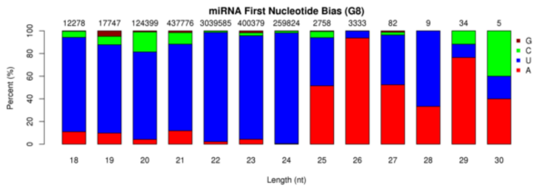
miRNA First Nucleotide Bias (G8)
12278 17747 124399 437776 3039585 400379 259824 2758 3333 82 9 34 5
Percent (%)
Length (nt)
G C U A

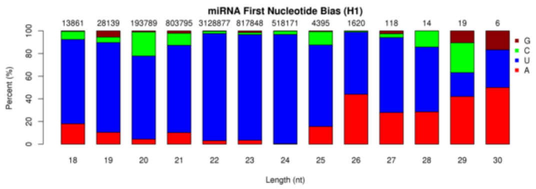
miRNA First Nucleotide Bias (H1)
13861 28139 193789 803795 3128877 817848 518171 4395 1620 118 14 19 6
Percent (%)
Length (nt)
G C U A

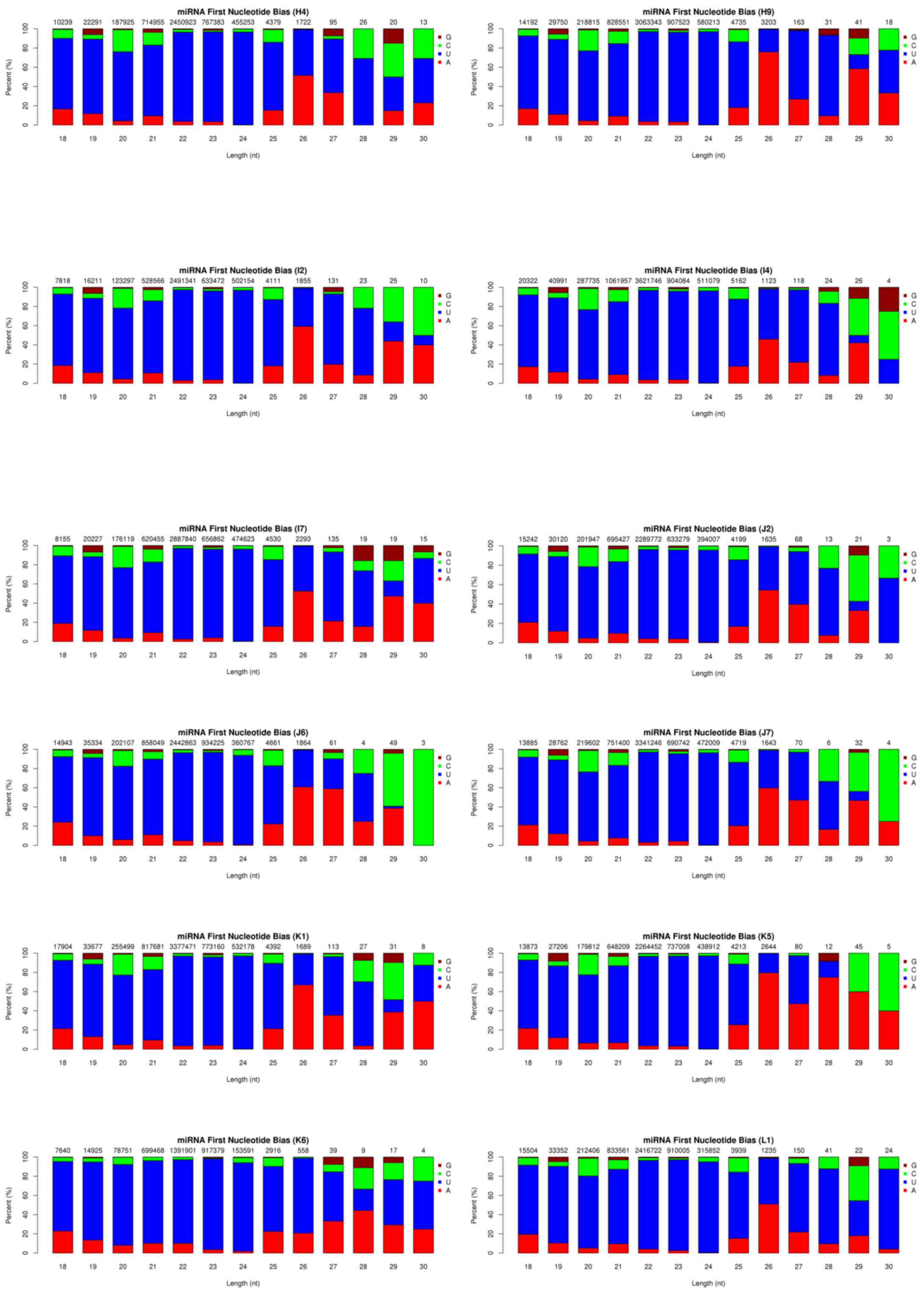
miRNA First Nucleotide Bias (H4)
10239 22291 187925 714955 2450923 767383 455253 4379 1722 95 26 20 13
miRNA First Nucleotide Bias (H9)
14192 29750 218815 828551 3063343 907523 580213 4735 3203 163 31 41 18
miRNA First Nucleotide Bias (I2)
7818 16211 123297 528566 2491341 633472 502154 4111 1855 131 23 25 10
miRNA First Nucleotide Bias (I4)
20322 40991 287735 1061957 3621746 904084 511079 5162 1123 118 24 26 4
miRNA First Nucleotide Bias (I7)
8155 20227 176119 620455 2887840 656862 474623 4530 2293 135 19 19 15
miRNA First Nucleotide Bias (J2)
15242 30120 201947 695427 2289772 633279 394007 4199 1635 68 13 21 3
miRNA First Nucleotide Bias (J6)
14943 35334 202107 858049 2442863 934225 360767 4661 1864 61 4 49 3
miRNA First Nucleotide Bias (J7)
13885 28762 219602 751400 3341246 690742 472009 4719 1643 70 6 32 4
miRNA First Nucleotide Bias (K1)
17904 33677 255499 817681 3377471 773160 532178 4392 1689 113 27 31 8
miRNA First Nucleotide Bias (K5)
13873 27206 179812 648209 2264452 737008 438912 4213 2644 80 12 45 5
miRNA First Nucleotide Bias (K6)
7640 14925 78751 699468 1391901 917379 153591 2916 558 39 9 17 4
miRNA First Nucleotide Bias (L1)
15504 33352 212406 833561 2416722 910005 315852 3939 1235 150 41 22 24
Percent (%)
Length (nt)
G
C
U
A

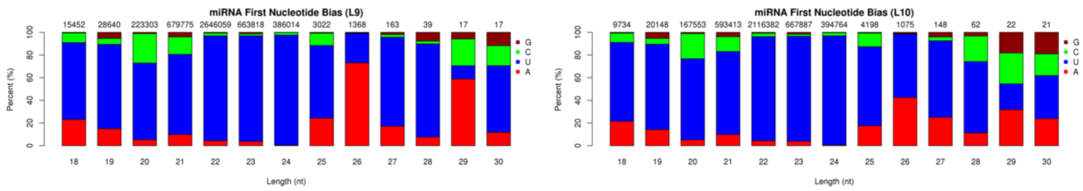

Supplementary Figure 5. Known miRNA first base preference of 18~30nt in length

The abscissa is the length of the miRNA, and the ordinate is the percentage of A/U/C/G in the first base of the miRNA in this length (the value above the histogram is the total number of miRNAs of this length)

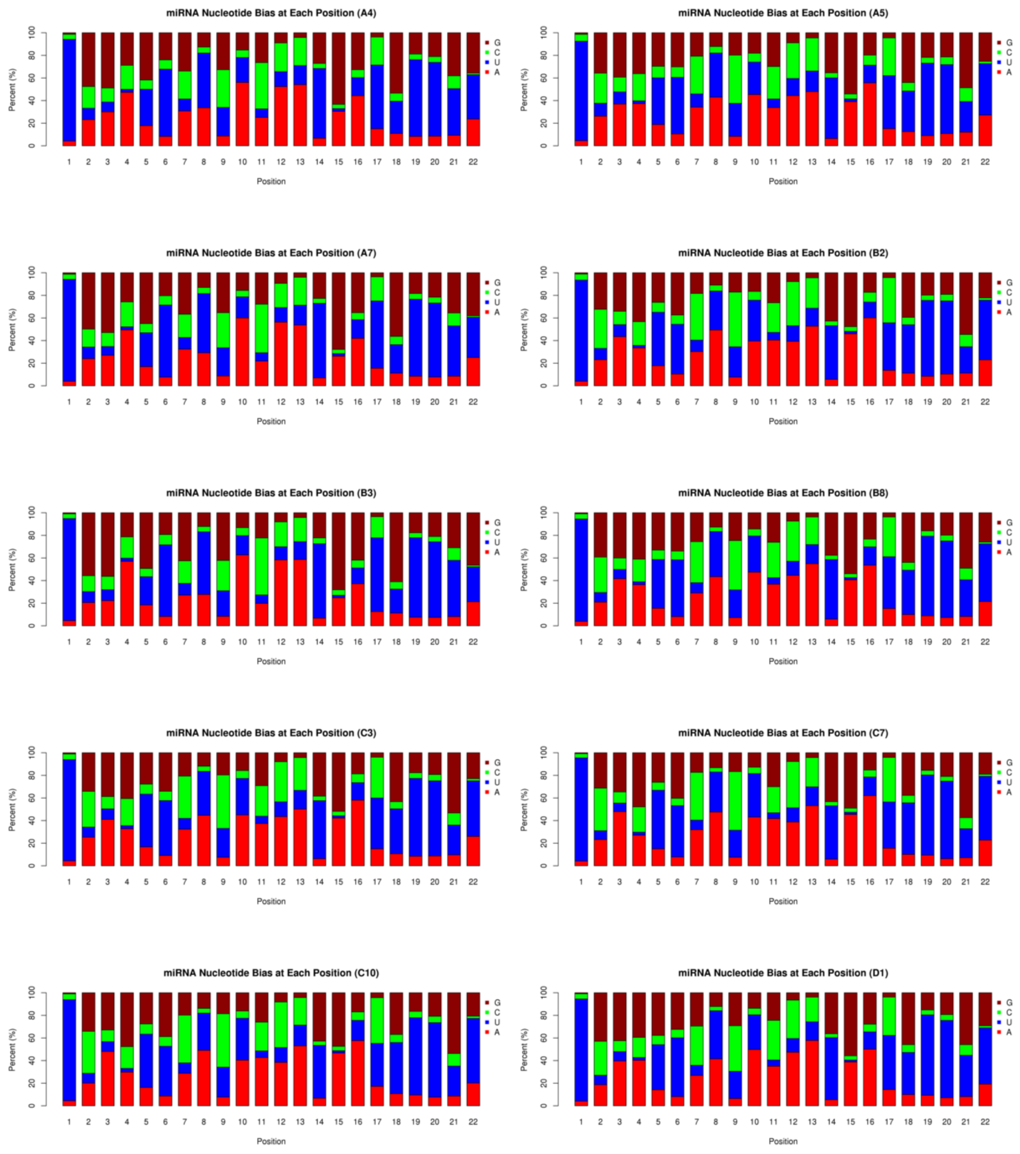

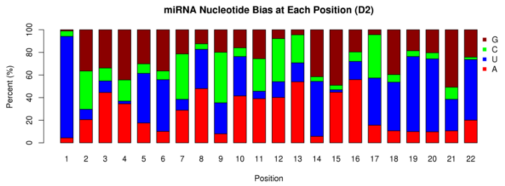
miRNA Nucleotide Bias at Each Position (D2)
Percent (%)
Position
G
C
U
A

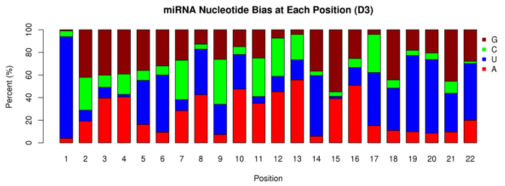
miRNA Nucleotide Bias at Each Position (D3)
Percent (%)
Position
G
C
U
A

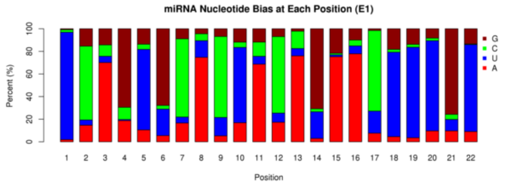
miRNA Nucleotide Bias at Each Position (E1)
Percent (%)
Position
G
C
U
A

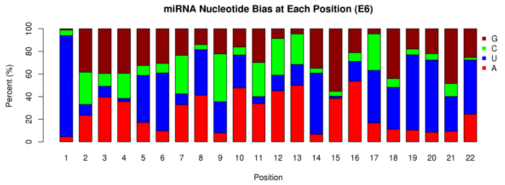
miRNA Nucleotide Bias at Each Position (E6)
Percent (%)
Position
G
C
U
A

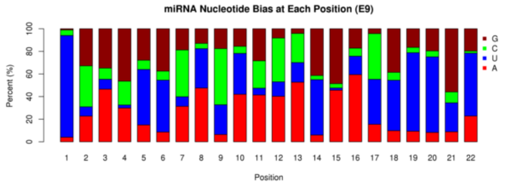
miRNA Nucleotide Bias at Each Position (E9)
Percent (%)
Position
G
C
U
A

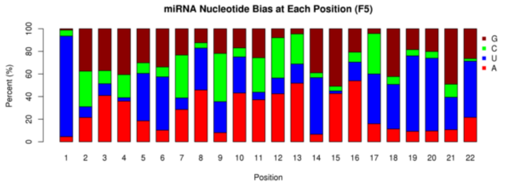
miRNA Nucleotide Bias at Each Position (F5)
Percent (%)
Position
G
C
U
A

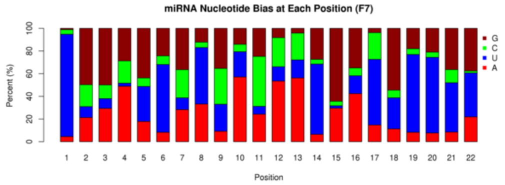
miRNA Nucleotide Bias at Each Position (F7)
Percent (%)
Position
G
C
U
A

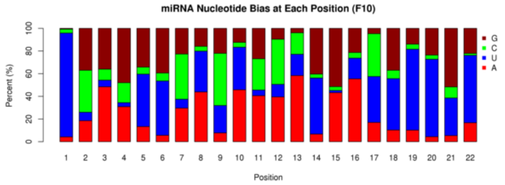
miRNA Nucleotide Bias at Each Position (F10)
Percent (%)
Position
G
C
U
A

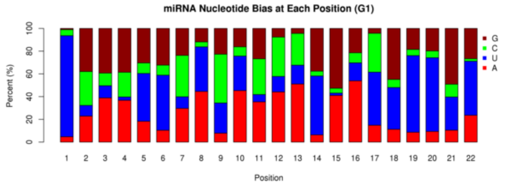
miRNA Nucleotide Bias at Each Position (G1)
Percent (%)
Position
G
C
U
A

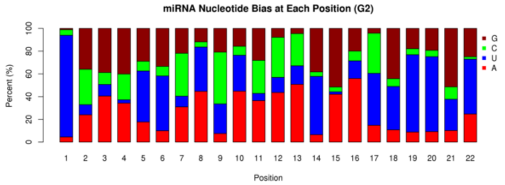
miRNA Nucleotide Bias at Each Position (G2)
Percent (%)
Position
G
C
U
A

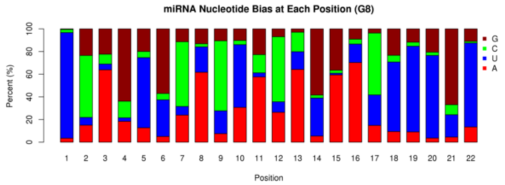
miRNA Nucleotide Bias at Each Position (G8)
Percent (%)
Position
G
C
U
A

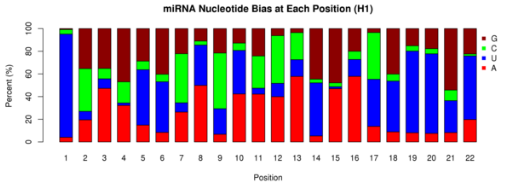
miRNA Nucleotide Bias at Each Position (H1)
Percent (%)
Position
G
C
U
A

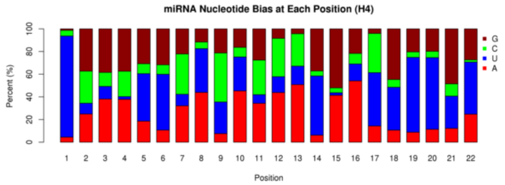
miRNA Nucleotide Bias at Each Position (H4)
Percent (%)
Position
G
C
U
A

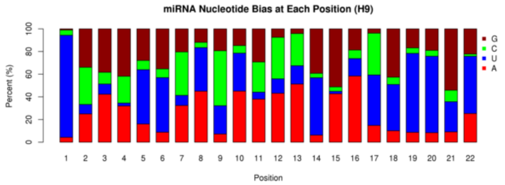
miRNA Nucleotide Bias at Each Position (H9)
Percent (%)
Position
G
C
U
A

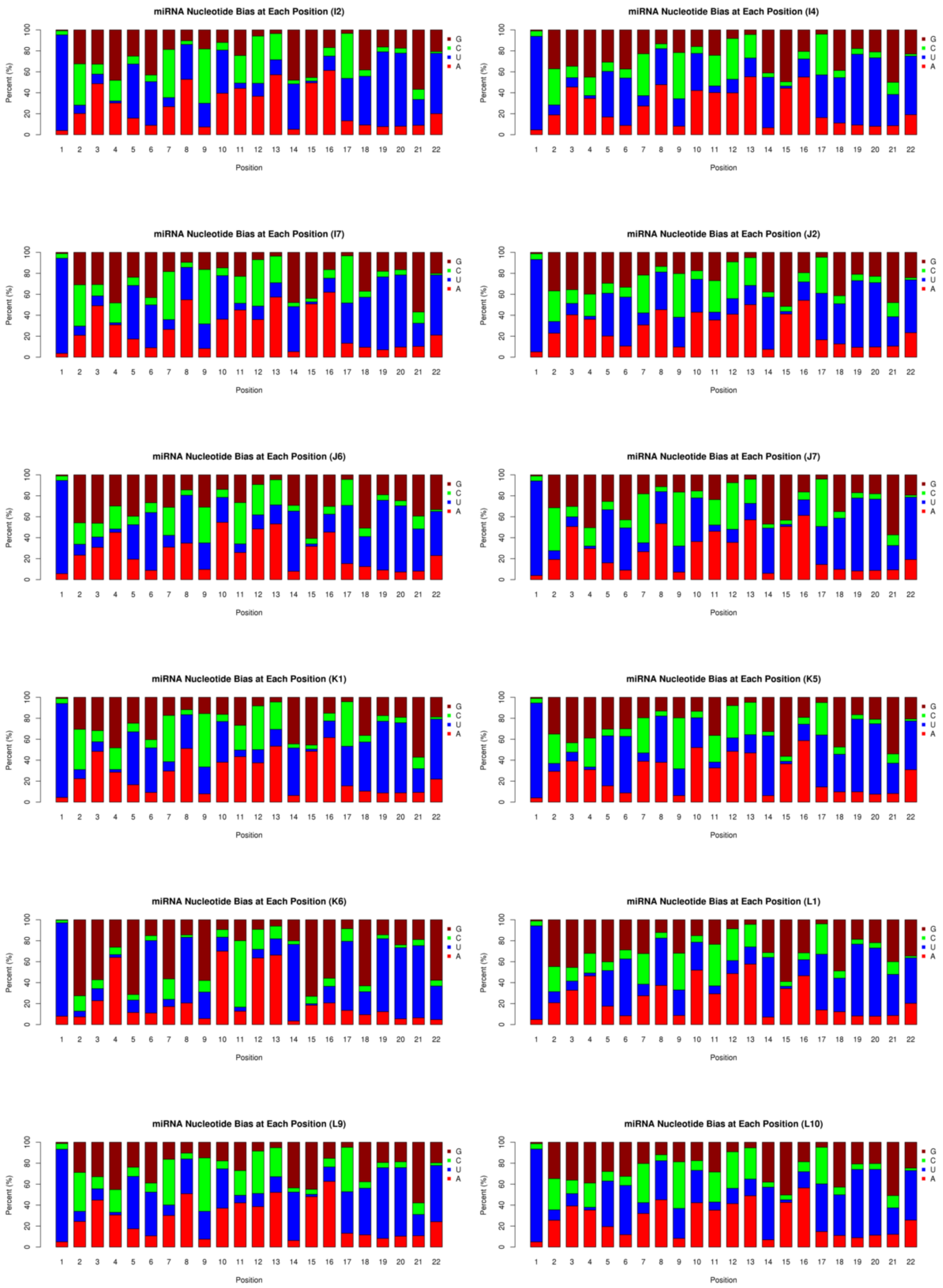

Supplementary Figure 6. Known miRNA base preference

The abscissa is the base position of the miRNA, and the ordinate is the percentage of bases A/U/C/G in the miRNA at this position.

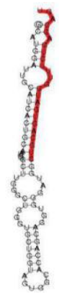

Supplementary Figure 7. Secondary structure of a partially predicted Novel miRNA.

The entire sequence is the miRNA precursor, and the red highlight is the mature sequence.

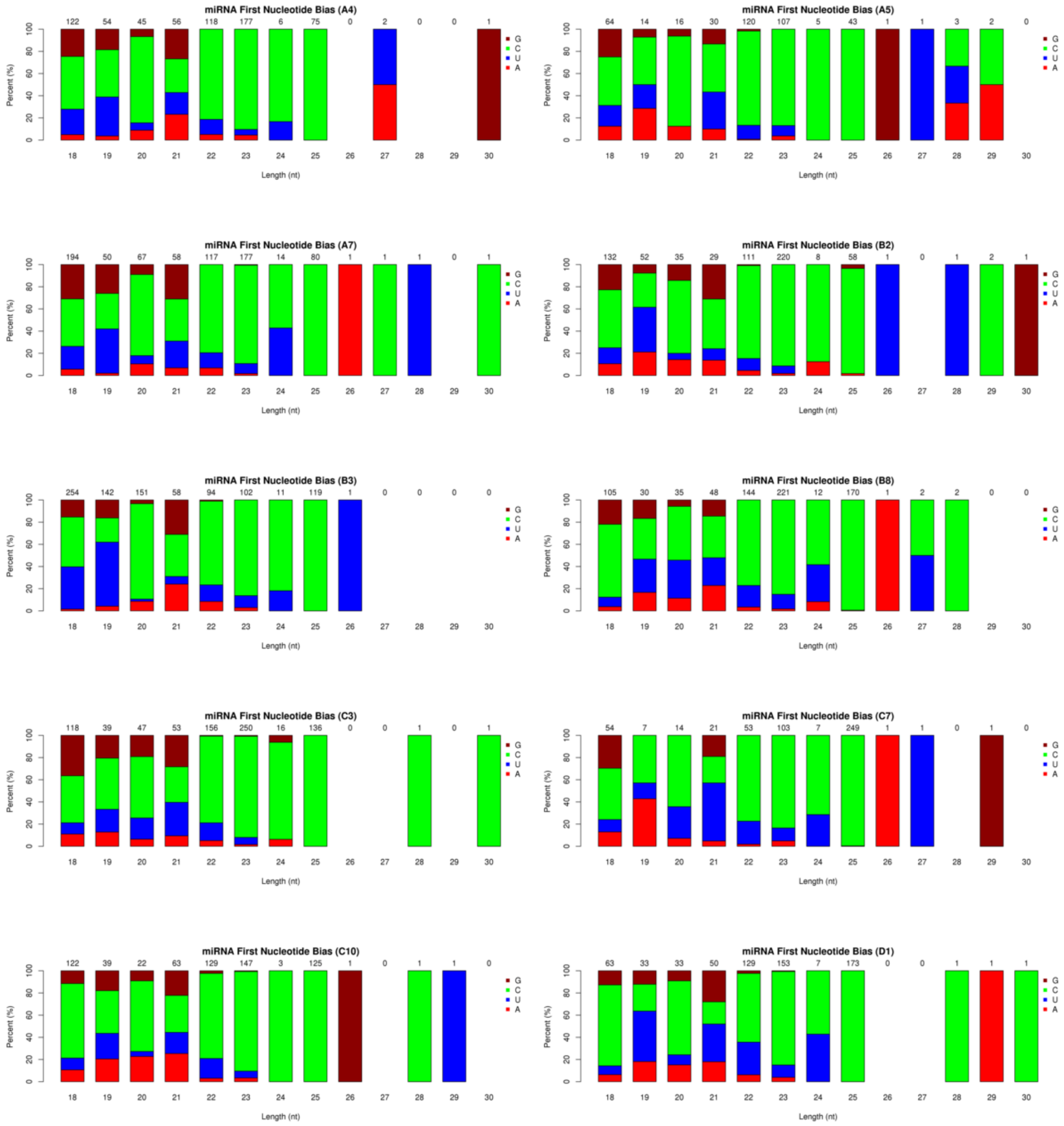

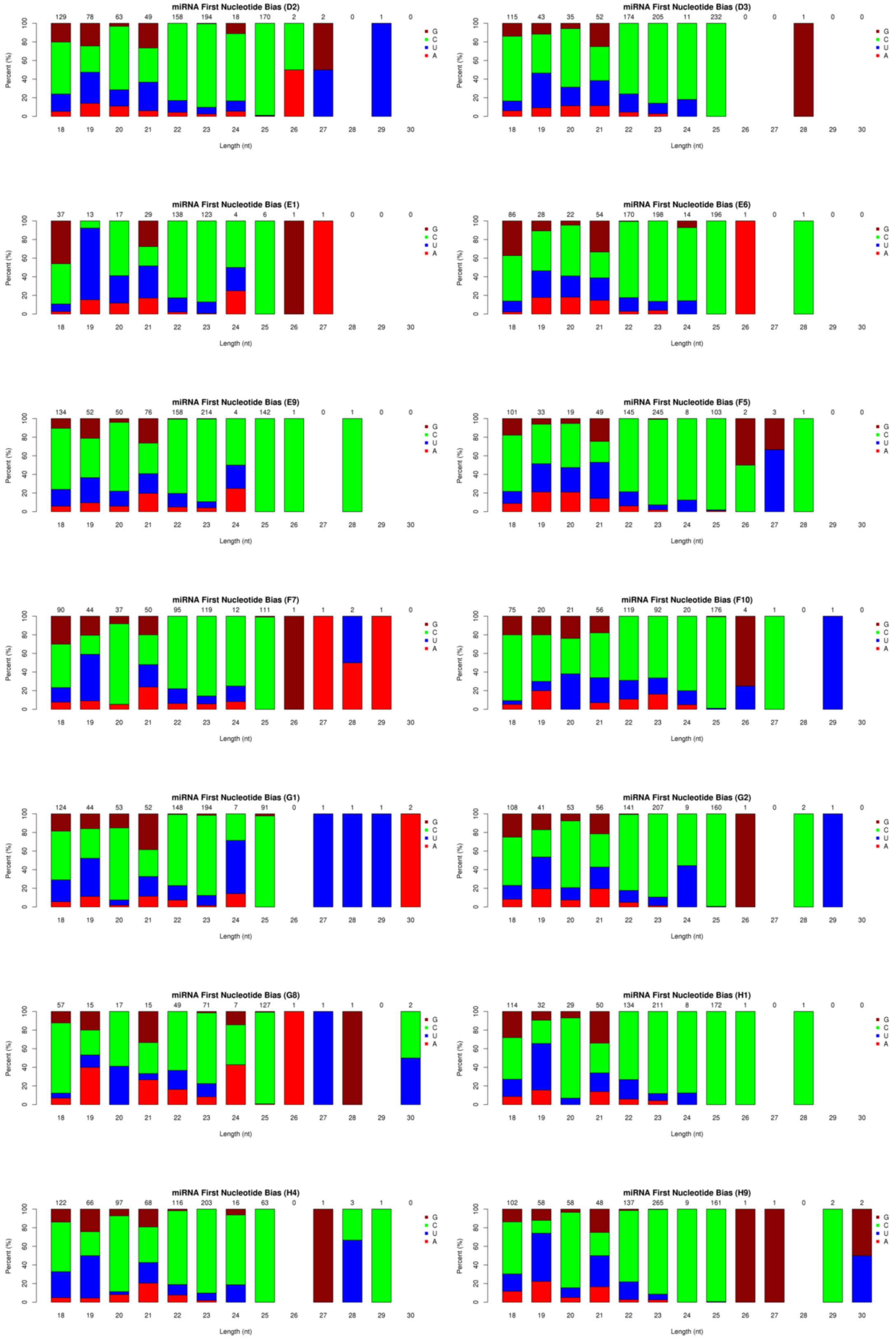
miRNA First Nucleotide Bias (D2)
miRNA First Nucleotide Bias (D3)
miRNA First Nucleotide Bias (E1)
miRNA First Nucleotide Bias (E6)
miRNA First Nucleotide Bias (E9)
miRNA First Nucleotide Bias (F5)
miRNA First Nucleotide Bias (F7)
miRNA First Nucleotide Bias (F10)
miRNA First Nucleotide Bias (G1)
miRNA First Nucleotide Bias (G2)
miRNA First Nucleotide Bias (G8)
miRNA First Nucleotide Bias (H1)
miRNA First Nucleotide Bias (H4)
miRNA First Nucleotide Bias (H9)
Percent (%)
Length (nt)
G
C
U
A

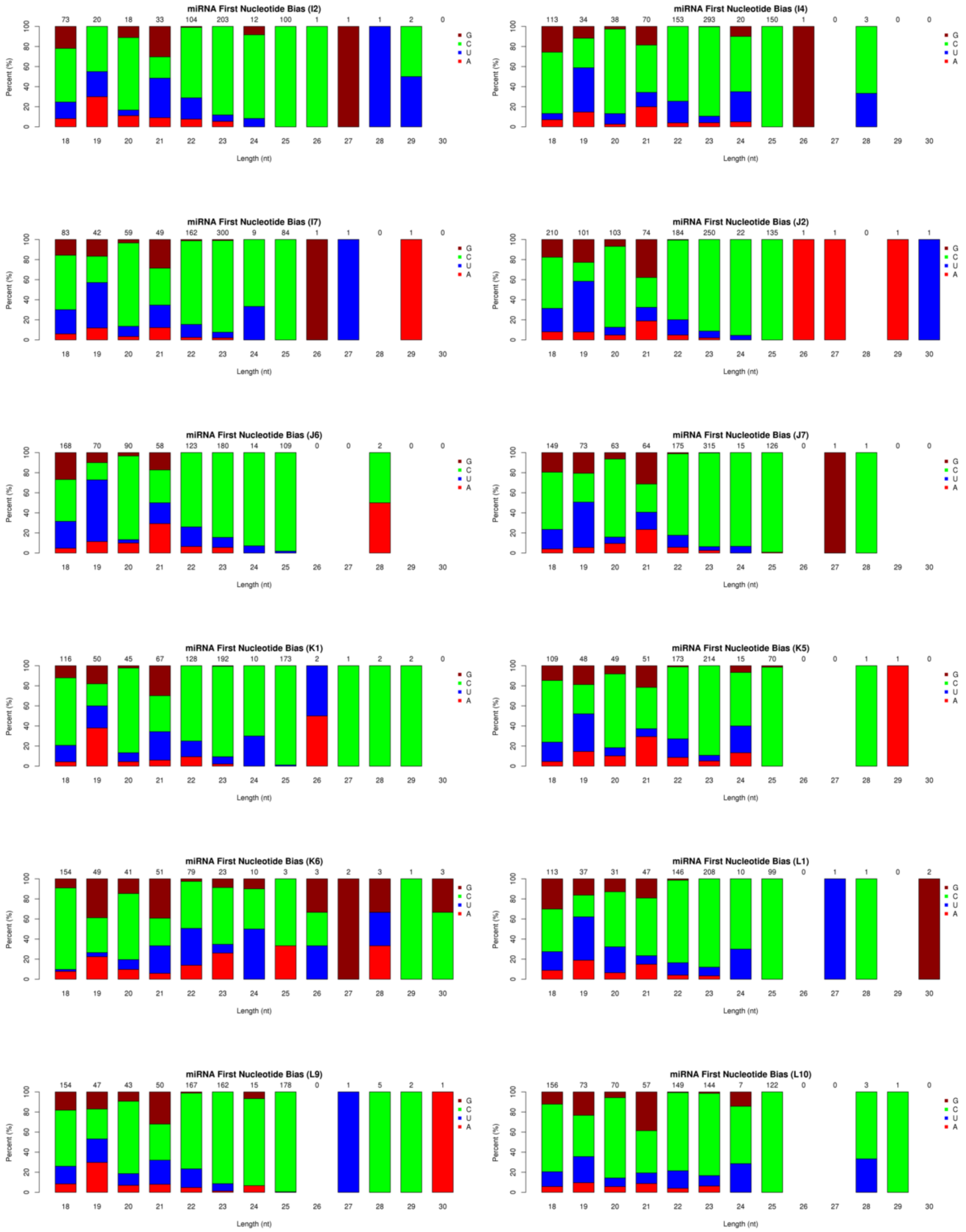

Supplementary Figure 8. First base preference of novel miRNAs with lengths from 18 to 30 nt.

The abscissa is the length of the miRNA, and the ordinate is the percentage of A/U/C/G in the first base of the length sRNA (the value above the bar graph is the total number of miRNAs of the length)

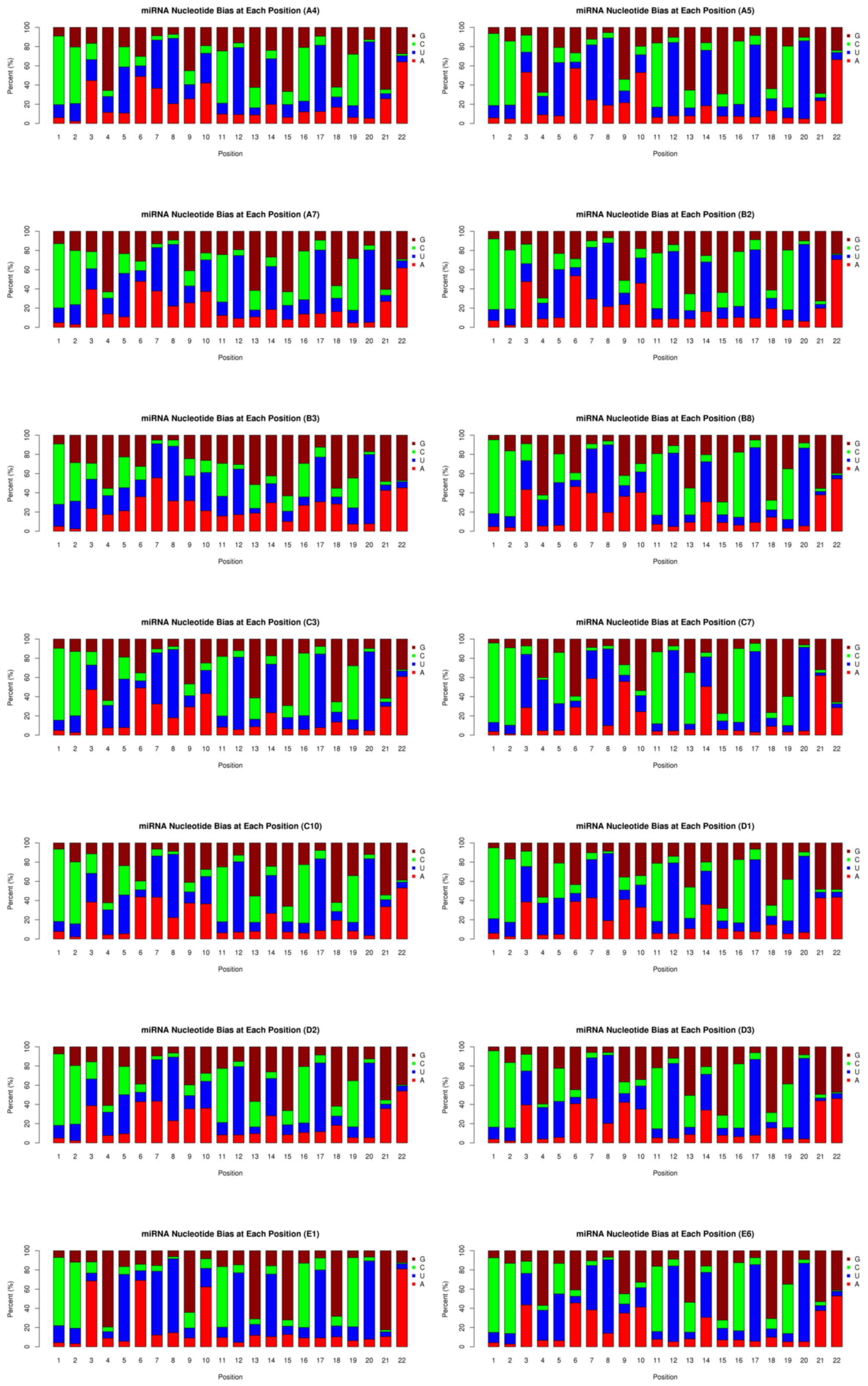
miRNA Nucleotide Bias at Each Position (A4)
miRNA Nucleotide Bias at Each Position (A5)
miRNA Nucleotide Bias at Each Position (A7)
miRNA Nucleotide Bias at Each Position (B2)
miRNA Nucleotide Bias at Each Position (B3)
miRNA Nucleotide Bias at Each Position (B8)
miRNA Nucleotide Bias at Each Position (C3)
miRNA Nucleotide Bias at Each Position (C7)
miRNA Nucleotide Bias at Each Position (C10)
miRNA Nucleotide Bias at Each Position (D1)
miRNA Nucleotide Bias at Each Position (D2)
miRNA Nucleotide Bias at Each Position (D3)
miRNA Nucleotide Bias at Each Position (E1)
miRNA Nucleotide Bias at Each Position (E6)
Percent (%)
Position
G
C
U
A

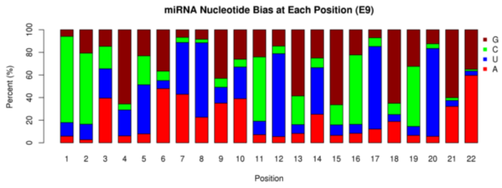
miRNA Nucleotide Bias at Each Position (E9)
Percent (%)
Position
G
C
U
A
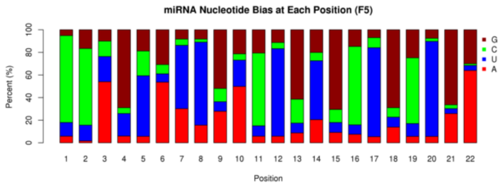
miRNA Nucleotide Bias at Each Position (F5)
Percent (%)
Position
G
C
U
A
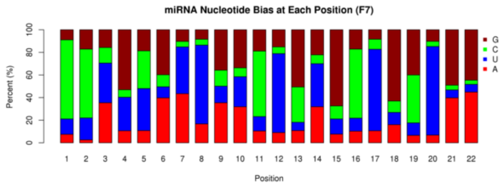
miRNA Nucleotide Bias at Each Position (F7)
Percent (%)
Position
G
C
U
A
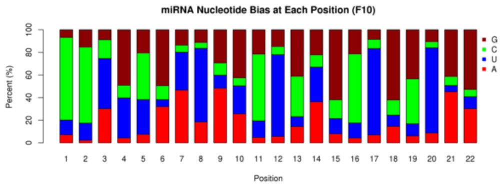
miRNA Nucleotide Bias at Each Position (F10)
Percent (%)
Position
G
C
U
A
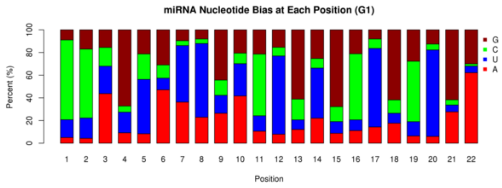
miRNA Nucleotide Bias at Each Position (G1)
Percent (%)
Position
G
C
U
A
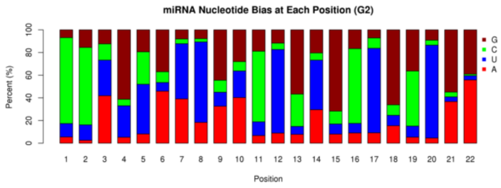
miRNA Nucleotide Bias at Each Position (G2)
Percent (%)
Position
G
C
U
A
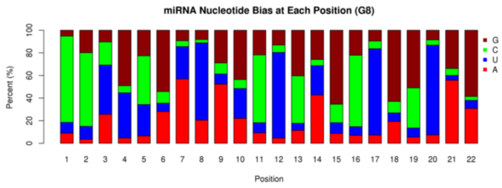
miRNA Nucleotide Bias at Each Position (G8)
Percent (%)
Position
G
C
U
A
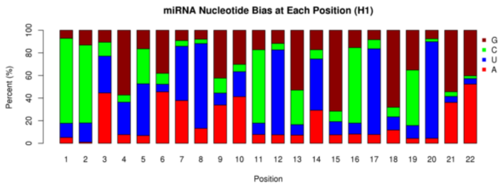
miRNA Nucleotide Bias at Each Position (H1)
Percent (%)
Position
G
C
U
A
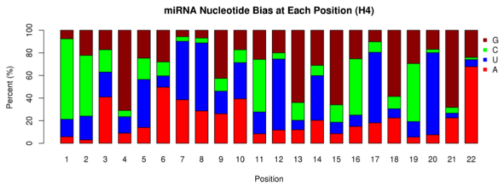
miRNA Nucleotide Bias at Each Position (H4)
Percent (%)
Position
G
C
U
A
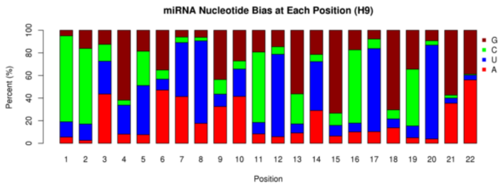
miRNA Nucleotide Bias at Each Position (H9)
Percent (%)
Position
G
C
U
A
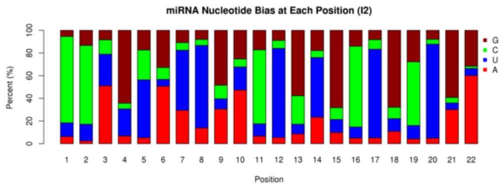
miRNA Nucleotide Bias at Each Position (I2)
Percent (%)
Position
G
C
U
A
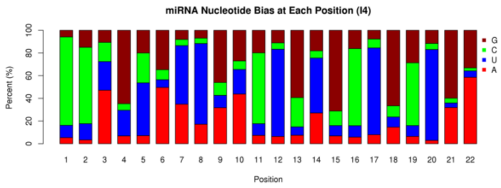
miRNA Nucleotide Bias at Each Position (I4)
Percent (%)
Position
G
C
U
A
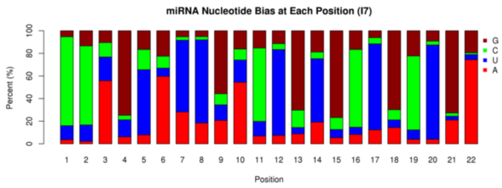
miRNA Nucleotide Bias at Each Position (I7)
Percent (%)
Position
G
C
U
A
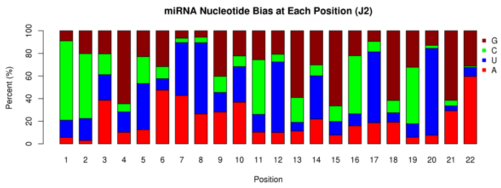
miRNA Nucleotide Bias at Each Position (J2)
Percent (%)
Position
G
C
U
A

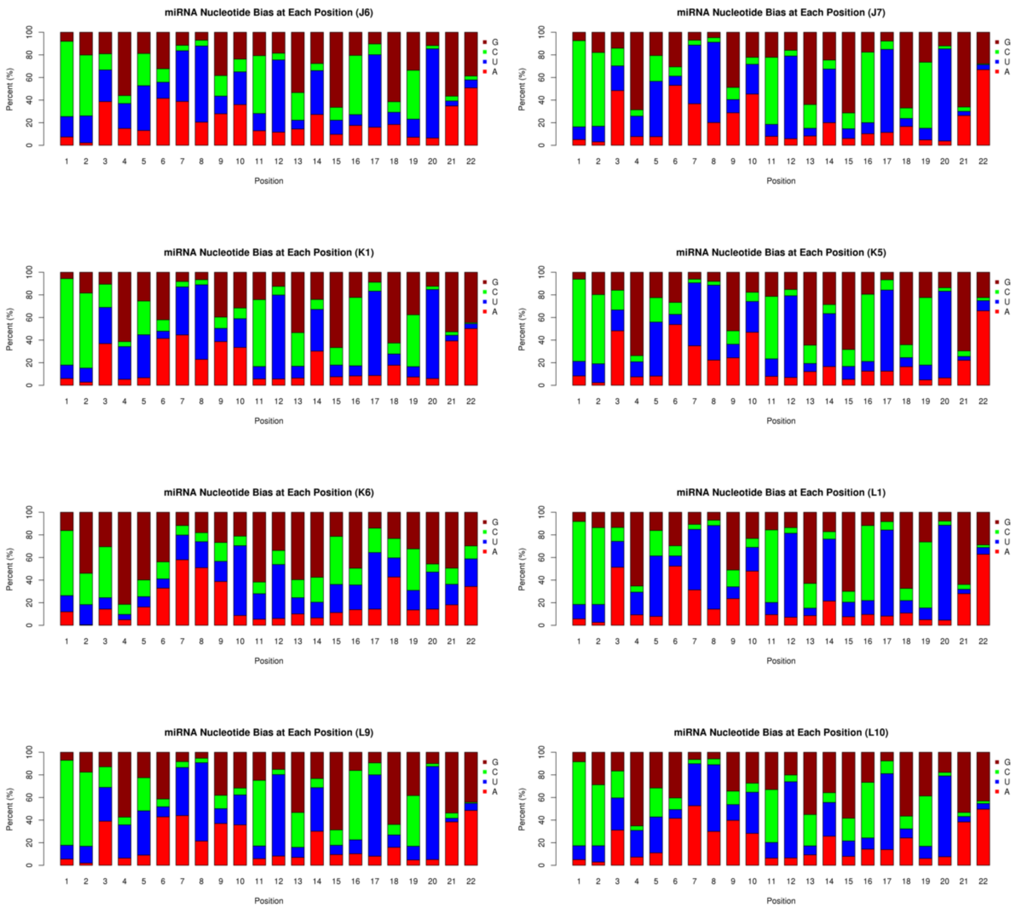

Supplementary Figure 9. Novel miRNA base preference.

The abscissa is the base position of the miRNA, and the ordinate is the percentage of bases A/U/C/G in the miRNA at this position

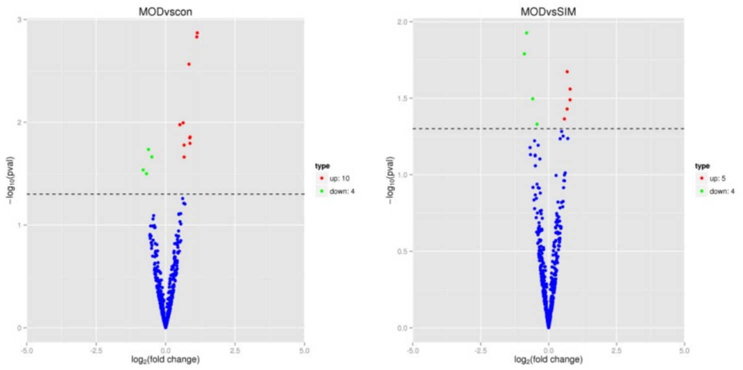

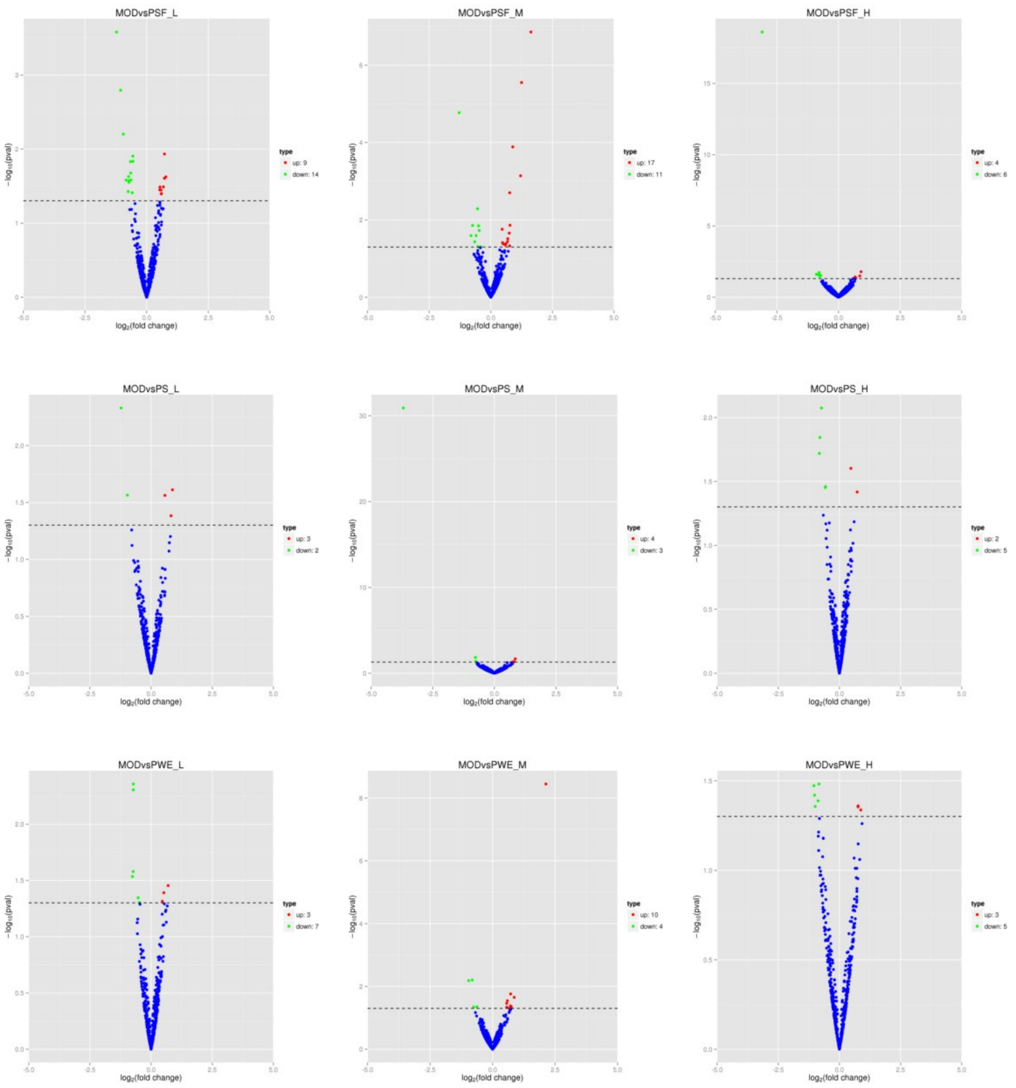

Supplementary Figure 10. Differential miRNA volcano map.

The abscissa represents the fold change in expression of miRNAs in different experimental groups/different samples, and the ordinate represents the statistical significance of the change in miRNA expression. The scatter points in the figure represent individual miRNAs, and the blue dots represent miRNAs with no significant differences. Red dots indicate significantly up-regulated differential miRNAs, and green dots indicate significantly down-regulated differential miRNAs

a

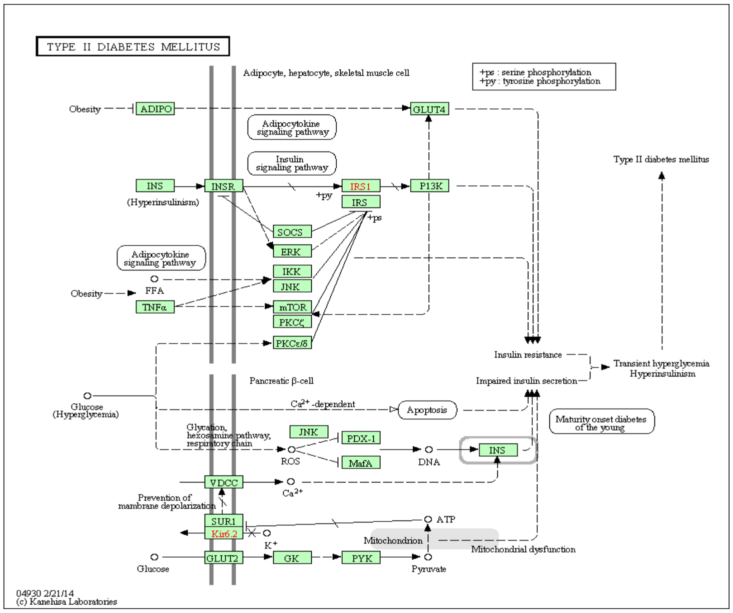
TYPE II DIABETES MELLITUS
Adipocyte, hepatocyte, skeletal muscle cell
+ps : serine phosphorylation
+py : tyrosine phosphorylation
Obesity
ADIPO
GLUT4
Adipocytokine signaling pathway
Insulin signaling pathway
Type II diabetes mellitus
INS
INSR
IRS1
+py
IRS
+ps
PI3K
(Hyperinsulinism)
SOCS
ERK
Adipocytokine signaling pathway
IKK
JNK
FFA
Obesity
TNFα
mTOR
PKCζ
PKCε/δ
Insulin resistance
Transient hyperglycemia
Hyperinsulinism
Pancreatic β-cell
Impaired insulin secretion
Glucose
(Hyperglycemia)
Ca2+ -dependent
Apoptosis
Maturity onset diabetes of the young
Glycation, hexosamine pathway, respiratory chain
JNK
PDX-1
ROS
MafA
DNA
INS
VDCC
Ca2+
Prevention of membrane depolarization
SUR1
Kir6.2
ATP
K+
Mitochondrion
Mitochondrial dysfunction
Glucose
GLUT2
GK
PYK
Pyruvate
04930 2/21/14
(c) Kanehisa Laboratories

b

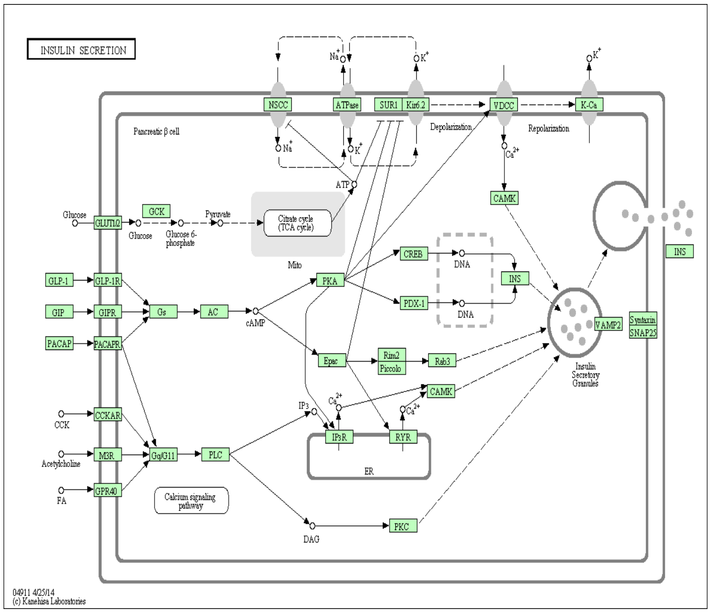
INSULIN SECRETION
Na+
K+
K+
NSCC
ATPase
SUR1
Kir6.2
VDCC
K-Ca
Pancreatic β cell
Depolarization
Repolarization
Na+
K+
Ca2+
ATP
CAMK
Glucose
GLUT1/2
GCK
Glucose
Glucose 6-phosphate
Pyruvate
Citrate cycle (TCA cycle)
Mito
INS
CREB
DNA
INS
GLP-1
GLP-1R
PKA
GIP
GIPR
Gs
AC
cAMP
PDX-1
DNA
VAMP2
Syntaxin
SNAP25
PACAP
PACAPR
Epac
Rim2
Piccolo
Rab3
Insulin Secretory Granules
CAMK
Ca2+
IP3
Ca2+
CCK
CCKAR
IP3R
RYR
Acetylcholine
M3R
Gq/G11
PLC
ER
FA
GPR40
Calcium signaling pathway
DAG
PKC
04911 4/25/14
(c) Kanehisa Laboratories

c

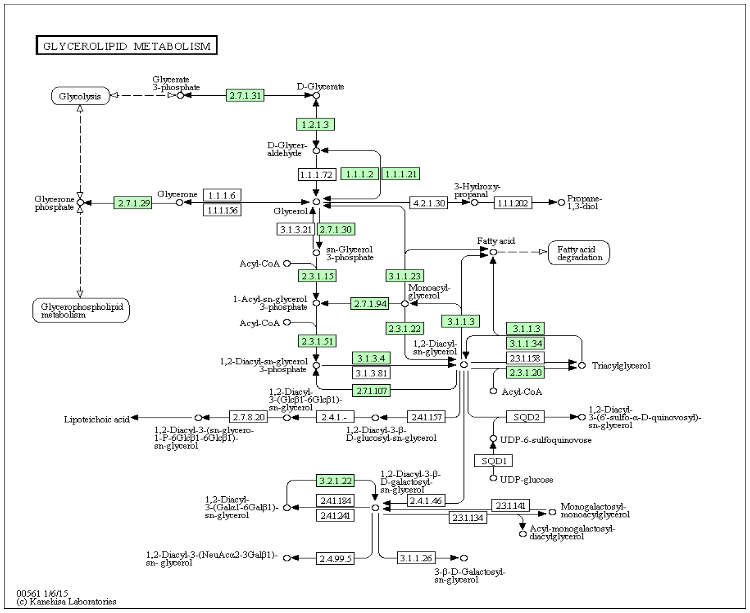

Supplementary Figure 11. Schematic diagram. **a** Schematic diagram of type 2 diabetes pathway b Schematic diagram of the insulin secretion pathway c Schematic diagram of glycerolipid metabolism pathway.
